# How Can Onchocerciasis Elimination in Africa Be Accelerated? Modeling the Impact of Increased Ivermectin Treatment Frequency and Complementary Vector Control

**DOI:** 10.1093/cid/cix1137

**Published:** 2018-06-01

**Authors:** Suzanne Verver, Martin Walker, Young Eun Kim, Grace Fobi, Afework H Tekle, Honorat G M Zouré, Samuel Wanji, Daniel A Boakye, Annette C Kuesel, Sake J de Vlas, Michel Boussinesq, Maria-Gloria Basáñez, Wilma A Stolk

**Affiliations:** 1 Department of Public Health, Erasmus MC, University Medical Center Rotterdam, The Netherlands; 2 Department of Pathobiology and Population Sciences and London Centre for Neglected Tropical Disease Research, Royal Veterinary College, Hatfield; 3 Department of Infectious Disease Epidemiology and London Centre for Neglected Tropical Disease Research, Imperial College London, United Kingdom; 4 Swiss Tropical and Public Health, Basel, Switzerland; 5 Independent Consultant, Yaoundé, Cameroon; 6 World Health Organization, Geneva, Switzerland; 7 Department of Microbiology and Parasitology, Faculty of Science, University of Buea, Cameroon; 8 Noguchi Memorial Institute of Medical Research, University of Ghana, Legon; 9 United Nations Children’s Fund/United Nations Development Programme/World Bank/World Health Organization Special Programme for Research and Training in Tropical Diseases, Geneva, Switzerland; 10 Institut de Recherche pour le Développement, Montpellier, France

**Keywords:** onchocerciasis, modeling, mass drug administration, ivermectin, elimination

## Abstract

**Background:**

Great strides have been made toward onchocerciasis elimination by mass drug administration (MDA) of ivermectin. Focusing on MDA-eligible areas, we investigated where the elimination goal can be achieved by 2025 by continuation of current practice (annual MDA with ivermectin) and where intensification or additional vector control is required. We did not consider areas hypoendemic for onchocerciasis with loiasis coendemicity where MDA is contraindicated.

**Methods:**

We used 2 previously published mathematical models, ONCHOSIM and EPIONCHO, to simulate future trends in microfilarial prevalence for 80 different settings (defined by precontrol endemicity and past MDA frequency and coverage) under different future treatment scenarios (annual, biannual, or quarterly MDA with different treatment coverage through 2025, with or without vector control strategies), assessing for each strategy whether it eventually leads to elimination.

**Results:**

Areas with 40%–50% precontrol microfilarial prevalence and ≥10 years of annual MDA may achieve elimination with a further 7 years of annual MDA, if not achieved already, according to both models. For most areas with 70%–80% precontrol prevalence, ONCHOSIM predicts that either annual or biannual MDA is sufficient to achieve elimination by 2025, whereas EPIONCHO predicts that elimination will not be achieved even with complementary vector control.

**Conclusions:**

Whether elimination will be reached by 2025 depends on precontrol endemicity, control history, and strategies chosen from now until 2025. Biannual or quarterly MDA will accelerate progress toward elimination but cannot guarantee it by 2025 in high-endemicity areas. Long-term concomitant MDA and vector control for high-endemicity areas might be useful.

## Teaser

We used 2 mathematical models, ONCHOSIM and EPIONCHO, to simulate future trends in microfilarial prevalence for 80 different settings under different future treatment scenarios, including mass drug administration and vector control, assessing whether they lead to elimination of onchocerciasis.

Onchocerciasis (river blindness), caused by the filarial nematode *Onchocerca volvulus* and transmitted by *Simulium* blackflies, occurs in many rural areas of sub-Saharan Africa. It can cause skin problems (itch, skin depigmentation, premature atrophy), visual impairment, and blindness, and is also associated with excess human mortality [1, 2] and possibly with epilepsy and nodding syndrome [2–4]. Despite large-scale, long-term control programs, the number of people infected in 2016 was still estimated at 15 million [5].

Control in Africa was initially based on vector control (VC) (the Onchocerciasis Control Programme in West Africa, 1974–2002), before community-wide mass drug administration (MDA) of ivermectin became the principal intervention. Ivermectin has some activity against adult worms [6], but predominantly reduces skin microfilarial loads through its microfilaricidal and embryostatic effects, thereby reducing symptoms and transmission [7]. When delivered annually, microfilarial prevalence declines slowly over time. For a long time, elimination of onchocerciasis through MDA was deemed impossible in Africa and therefore the African Programme for Onchocerciasis Control (APOC, 1995–2015) was defined as a morbidity control program [8]. In recent years, several African studies have indicated that elimination may be feasible by MDA, provided that sufficiently high and prolonged ivermectin treatment coverage is sustained [9–12].

The World Health Organization (WHO) now targets onchocerciasis elimination in selected African countries by 2020, and which APOC deemed achievable, and in the majority of African countries by 2025 [13, 14]. However, it is now recognized that annual MDA is unlikely to be sufficient to achieve these targets, and before its closure APOC proposed several “alternative treatment strategies” to accelerate elimination [15]. These include MDA at higher frequency and complementary VC through localized ground-based larviciding at *Simulium* breeding sites [15].

The relative impact of different alternative treatment strategies is uncertain. While controlled studies to compare different treatment strategies are not feasible, mathematical modeling can provide critical information for policy decisions. Two different models, ONCHOSIM and EPIONCHO [16], are in use to support policy making for onchocerciasis elimination programs [12, 17–27]. We used both models to simulate future trends in microfilarial prevalence and to assess whether elimination occurs under different future intervention strategies, including annual, biannual (ie, every 6 months), and quarterly (ie, every 3 months) MDA with ivermectin through 2025, with or without complementary VC. This is done for different settings defined by precontrol endemicity and MDA history, selected to reflect heterogeneity among the majority of endemic areas in APOC countries.

## METHODS

### Mathematical Models

Technical details for ONCHOSIM and EPIONCHO, references, and model comparison can be found in [16, 21, 22]. The parameter values used for EPIONCHO are the best combinations reported in Walker et al [22] from a full exploration of the parameter space. Supplementary Tables 2 and 3 of the Supplementary Materials list parameters for ONCHOSIM and EPIONCHO, respectively.

ONCHOSIM is an individual-based stochastic model, simulating transmission of onchocerciasis in a dynamic population of approximately 400 persons, representing a typical endemic village. The model “tracks” the life histories of individual male and female adult and populations of microfilariae within individual human hosts. Parasite transmission by a population of blackflies with the 3 larval (L1, L2, L3) stages within the vector is modeled deterministically, with seasonal variation in transmission defined by monthly biting rates (number of bites per person per month). The model accounts for age- and sex-dependent heterogeneity in exposure to blackfly bites and treatment compliance, with a flexible structure to model realistic distributions of adult worm lifespans [18, 28, 29].

EPIONCHO is a deterministic population-based transmission model that uses partial differential equations to describe changes with respect to time and host age in mean numbers of female adult worms per human host, microfilariae per milligram of skin, and (L1, L2, L3) larvae per blackfly [1, 16, 22]. EPIONCHO is based on a prototype presented in [30], extended to include age and sex structure of the human population [31] and temporal dynamics of microfilariae following ivermectin treatment [7]. The model incorporates age- and sex-specific patterns of exposure to blackfly bites [31] and the net effect on transmission of individual heterogeneity in exposure.

In both models, precontrol microfilarial prevalence levels are calibrated by adjusting the annual biting rates [16, 21, 22].

Responses to ivermectin treatment are modeled in a similar fashion in both models. In EPIONCHO, ivermectin is assumed to kill 98%–99% of microfilariae 1–2 months after treatment, to sterilize adult female worms temporarily, and to reduce cumulatively their capacity to produce microfilariae [7, 17]. In ONCHOSIM, 100% of microfilariae are killed instantaneously by treatment. Rates at which female worms resume microfilarial production are subtly different between the 2 models [16, 21, 32]. Both models assume that children <5 years of age are excluded and that 5% of the remaining population never participates in MDA (ie, systematic nonadherers) [33]. EPIONCHO partitions the population into 4 treatment groups: a full adherence group that takes treatment every round; 2 semiadherent groups that take treatment every other round alternately; and a systematically nonadherent group that never takes treatment. In ONCHOSIM, participation of individuals in MDA rounds is driven by a probability that depends on age and sex, and a parameter governing a personal inclination to adhere to MDA.

### Simulated Settings and Treatment Scenarios

Simulations were done for 80 different settings, defined by the assumed precontrol endemicity level (precontrol microfilarial prevalence in ≥5-year-olds of 40%, 50%, 60%, 70%, or 80%) and history of control (0, 5, 10, 15, or 20 years of annual MDA or 5 years of biannual MDA, with coverage of 50%, 65%, or 80%), to capture a large fraction of the heterogeneity across African endemic areas. Settings were assumed to be eligible for MDA (low-endemic onchocerciasis areas with loiasis coendemicity are therefore not considered; these areas require alternative interventions). For each setting, we evaluated whether continuation of MDA was needed for elimination and whether elimination could be achieved within 7 years by continuing current practice of annual MDA or switching to one of the alternative treatment strategies (Table 1), with or without complementary VC.

**Table 1.** Elements of Future Treatment Strategies

Type	Strategy Code	Explanation	Remarks
Future MDA	A65, A80	7 y of aMDA with ivermectin, starting in 2019 with the last treatment given in 2025, covering 50%, 65%, or 80% of the population, respectively. aMDA is provided in month 6, just before peak transmission season.	aMDA is not considered in settings with a history of bMDA; for settings with a history of control, we do not consider strategies involving a reduction in coverage or treatment frequency.
	B65, B80	As above, but with bMDA. Treatment is provided in months 6 and 12 (14 treatment rounds over 7 y).	
	Q65, Q80	As above, but with quarterly MDA. Treatment is provided in months 3, 6, 9, and 12 (28 treatment rounds).	
Future VC	VC5, VC15	VC through ground-based larviciding, continued for 5 or 15 y, respectively. VC starts 2 y later than future MDA (from January 2021 onward) to allow for necessary preparations; VC ends in the same year as MDA if continued for only 5 y, and 10 y later with the 15-y duration.	
Evaluation	E	Elimination is predicted to have been achieved with the current strategy used to 2018; evaluation should be scheduled, and until then, the current strategy should be continued (with at least 65% treatment coverage).	Only considered in settings with a history of annual or bMDA, to check whether additional interventions are needed at all.

Abbreviations: aMDA, annual mass drug administration; bMDA, biannual mass drug administration; MDA, mass drug administration; VC, vector control.

The 7-year MDA duration (2019–2025) was chosen to align with the 2025 elimination target year and the preparation period required to implement a change in MDA strategy. Complementary VC was assumed to be implemented locally by regular (weekly or monthly) ground larviciding of blackfly breeding sites [34, 35]. This may require a longer preparation period, for identification of breeding sites and *Simulium* species, determination of larvicide efficacy and optimal dosage, and so forth [34]. Therefore, the start of VC was assumed to begin in 2021. We considered different durations of VC: 5 years (assuming that VC stops in 2025, the same stop year as MDA) or 15 years (ie, spanning the estimated average reproductive life span of adult worms). The effect of VC was modeled as a constant 70% reduction in biting rates [35].

### Identification of Preferred Strategy

We identified the preferred strategy to achieve elimination by 2025 for each model. When the models predicted that ≥2 strategies could lead to elimination, the more feasible strategies were given preference: (1) Annual MDA with higher coverage was preferred to biannual or quarterly MDA with lower coverage; and (2) strategies with shorter VC were preferred to strategies with longer VC (except in cases when quarterly MDA was indicated; scenarios with annual or biannual MDA plus VC were given as equal alternatives to quarterly MDA). For settings with a history of control, we did not consider a reduction in treatment frequency (from biannual to annual) or coverage.

### Simulations and Analysis

The stochasticity in ONCHOSIM means that repeated simulations for the same scenario give slightly different results, sometimes ending in elimination and sometimes in recrudescence. Therefore, each scenario was run 1000 times, and we calculated the average microfilarial prevalence and the percentage of simulation runs resulting eventually in zero microfilarial prevalence. Elimination was said to occur if ≥99% of simulations resulted in zero microfilarial prevalence 50 years after cessation of MDA. The 50-year time horizon permitted robust assessment of whether the parasite population became extinct or recrudesced because worm and microfilarial prevalence do not necessarily have to be zero when interventions are stopped.

For EPIONCHO, a single (deterministic) simulation was run for each scenario to determine the average trend in microfilarial prevalence over time and to identify whether elimination was reached 50 years after cessation of MDA. Elimination was considered achieved if the mean number of all parasite stages tended terminally to zero. This determines numerically whether the transmission breakpoint had been crossed [22].

## RESULTS

**Figure 1.**
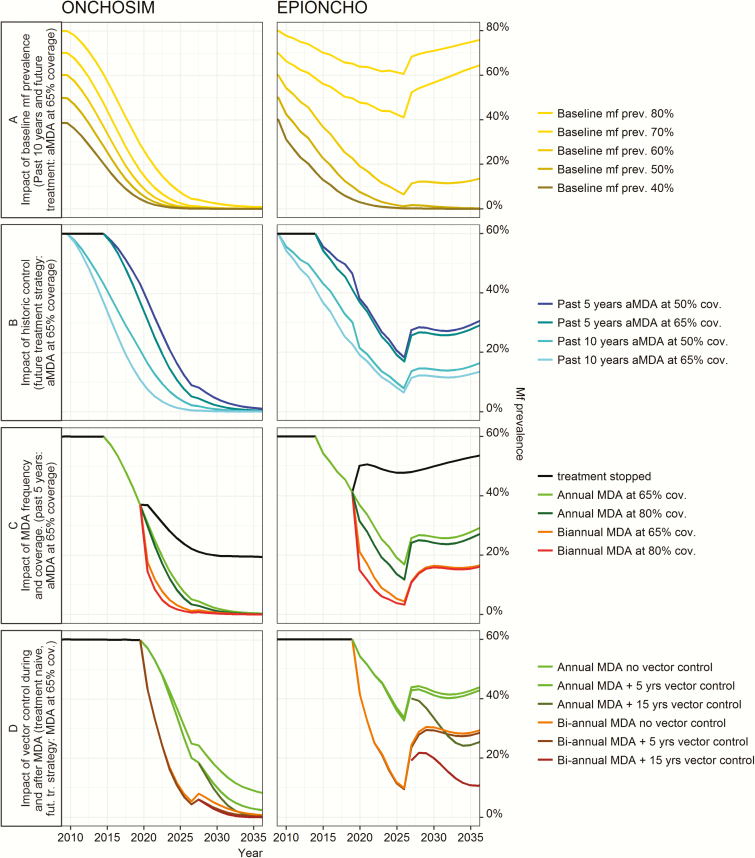
Illustrative predicted trends in onchocerciasis microfilarial prevalence in different settings of endemicity with different past and future interventions. For full set of graphs, see Supplementary Data 2 and 3. Microfilarial prevalence projected by ONHCOSIM represents the mean per 1000 model runs. The projections are deterministic for EPIONCHO. Labeled future intervention strategies were simulated from 2019 to 2025 (vector control 15 years has vector control but not mass drug administration applied until 2035). *A*, Precontrol (baseline) microfilarial prevalence. *B–D*, 60% precontrol microfilarial prevalence. *D*, No history of control. Abbreviations: aMDA, annual mass drug administration; cov., coverage; fut. tr., future treatment; MDA, mass drug administration; mf, microfilariae; prev. = prevalence.

Figure 1 shows predicted trends in microfilarial prevalence for selected settings and treatment scenarios for both models during interventions. Prospects for achieving elimination by 2025 strongly depend on precontrol endemicity level (Figure 1A) and history of control (Figure 1B). Both factors determine the model-predicted residual microfilarial prevalence that remains now in 2017 and after stopping MDA in 2025. The greatest reductions in microfilarial prevalence are achieved when MDA coverage is high or treatment is given frequently (Figure 1C), whereas adding VC during MDA has little impact on microfilarial prevalence dynamics (Figure 1D, compare dashed and solid lines). Continuation of VC after cessation of MDA, however, can help to prevent recrudescence after cessation of MDA.

Figure 1 also shows that the 2 models give very different predictions. In Figure 1A, for example, ONCHOSIM predicts a decline in microfilarial prevalence to <5% even in high-precontrol-prevalence settings, whereas the EPIONCHO-predicted decline is much lower and the modest reduction in prevalence in high-endemicity settings leads to fast recrudescence after stopping annual MDA in 2025.

Table 2 identifies for each model the preferred strategy to achieve elimination after stopping MDA in 2025. This figure clearly shows that more efforts are needed in settings with higher precontrol prevalence or a shorter or less intensive history of control. Some of the settings with a history of ≥10 years of MDA are predicted to have achieved the elimination target already, and the model preferred strategy for such settings is to proceed to evaluation (continuing the current strategy until that time, ensuring high coverage and preventing systematic nonadherence).

**Table 2.**
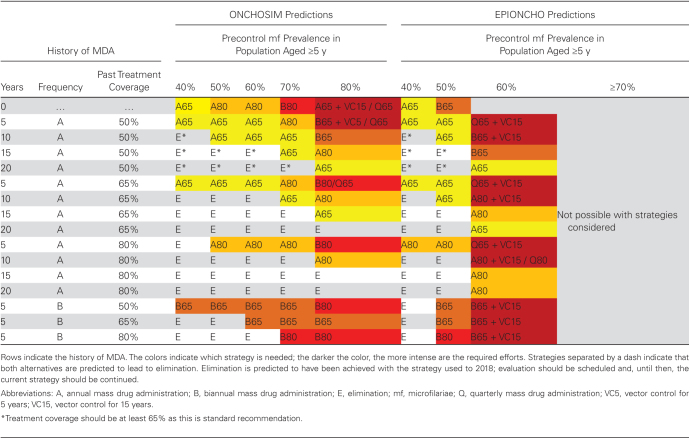
Strategies Predicted to Achieve Onchocerciasis Elimination: Preferred Strategy Required From 2019 With Maximum 7 Years of Mass Drug Administration to Achieve Elimination of Onchocerciasis as Predicted by the Transmission Models ONCHOSIM and EPIONCHO

For most low-endemic settings (40%–50% precontrol microfilarial prevalence), both models predict that the 2025 elimination target can be achieved by continuing the current strategy of annual MDA, if not achieved already. Only few of these settings are predicted to require an increase in coverage or treatment frequency (more often with EPIONCHO than with ONCHOSIM).

The predictions for settings with ≥60% precontrol prevalence vary strongly between models. ONCHOSIM suggests that elimination is achievable in most settings with MDA alone, although this might require an increase in treatment frequency or coverage. Only in settings with 80% precontrol microfilarial prevalence and no or only 5 years of past MDA, quarterly treatment or addition of VC would be needed. EPIONCHO, however, suggests that such intensive strategies are already required in settings with 60% precontrol prevalence, and predicts that elimination cannot be achieved with 7 years of MDA with any of the strategies examined in settings with ≥70% microfilarial prevalence.

Detailed simulation results by setting (defined by precontrol microfilarial prevalence and history of control), showing the trends under the various alternative treatment strategies, are shown for each model in the Supplementary Materials.

## DISCUSSION

We show that precontrol endemicity levels (microfilarial prevalence) and control histories (when MDA began and how effectively it was implemented) drive the intervention strategies needed for onchocerciasis elimination by 2025 or thereafter. For many low-endemic settings (40%–50% precontrol microfilarial prevalence), continuation of the current strategy of annual MDA may be sufficient to achieve elimination by 2025 if not achieved already. By contrast, many settings with higher precontrol levels may need an intensified MDA strategy (increase in coverage, increase in frequency) and sometimes complementary VC, particularly those with a short history of control.

### Uncertainty

In general, ONCHOSIM’s predictions regarding elimination prospects are much more optimistic than EPIONCHO’s, which accords with our previous model comparison study [18], and remained after revision of the EPIONCHO model [22]. We have identified many factors that contribute to the difference. Differences in microfilarial prevalence dynamics during the intervention are strongly influenced by the different assumptions made by the models on the sensitivity of the skin snip. ONCHOSIM assumes a random (Poisson) distribution of microfilariae within the skin, implying high sensitivity, whereas EPIONCHO models an aggregated or patchy (negative binomial) distribution, implying low sensitivity, especially in low-intensity infections [36]. The models also differ in their representation of the relationship between microfilarial prevalence and vector biting rate. In EPIONCHO, high precontrol microfilarial prevalence is associated with a much greater underlying intensity of transmission (vector biting rate) than ONCHOSIM [22], making elimination more difficult to achieve. Moreover, ONCHOSIM considers transmission dynamics in a single community of about 400 individuals and chance elimination by stochastic processes may frequently occur, whereas the population-based EPIONCHO implicitly represents a very large population, which helps to stabilize transmission (and does not include stochastic/chance elimination). Moreover, EPIONCHO takes account of several density-dependent processes, which are not considered in ONCHOSIM, that enhance the efficiency of transmission when parasite intensity in the population becomes low. Elimination in ONCHOSIM is accelerated by the assumption that older worms produce fewer microfilariae than young worms [37], which is not considered in EPIONCHO. Moreover, neither model includes external forces of infection from neighboring communities. In reality, infection dynamics will be influenced by movement of infected humans, flies, or changes in demographic, geographic, and environmental conditions. Some of the factors highlighted above could make ONCHOSIM too optimistic or EPIONCHO too pessimistic. The ranges presented in Table 2 give a good indication of real uncertainties, and the truth may lie between the predictions of these 2 models.

### Choosing a Strategy

Our work may help onchocerciasis elimination programs to choose the most appropriate intervention strategy to achieve the time-bounded elimination targets. Within a treatment area, there is usually variation among community endemicities, achieved coverage, and the fraction of the population that never participates in mass treatment. Programs should ideally choose a strategy that is sufficient to achieve the elimination target in all settings within the larger area. Yet, the choice is often driven by other factors, including logistical feasibility, budgetary, and resource constraints. We have based the selection of strategies in Table 2 on the likely ease of implementation, but not on cost-effectiveness analyses. Previous modeling has shown that treating biannually or even quarterly, although increasing the total number of treatment rounds compared to annual MDA [21], can achieve cost-savings by shortening program duration, particularly in highly endemic settings [27].

We have shown that localized VC can sometimes help to achieve elimination targets or can help to maintain the gains after cessation of MDA, especially in very highly endemic areas. It may also be an attractive option to control the nuisance from biting blackflies. In practice, VC through larviciding might be implemented just before and during the blackfly breeding season. More research on the feasibility and cost-effectiveness of ground-based larviciding is needed, as are submodels that more accurately reflect the effect of larviciding on blackfly population dynamics.

### Evaluation

Implementation of a selected strategy does not guarantee success, due to uncertainty about local endemicity, past and future (therapeutic) coverage patterns, and geographic coverage. If MDA coverage is lower or the fraction of systematic nonadherers higher than assumed in our simulations, the effectiveness of interventions will likely deviate from the model predictions [33]. Decisions to stop intervention activities need to be based on field evaluations including transmission assessment (measuring infection or past exposure in humans and blackflies) across intervention areas, to test whether residual infection levels in human and blackfly infectivity are below thresholds associated with minimal risk of recrudescence.

Residual onchocerciasis infection may persist for some time, even when elimination may ultimately be achieved. Some strategies that lead to elimination 50 years after stopping MDA are associated with relatively high microfilarial prevalence (~10%) 1 year after the last MDA round (Figure 1, rows 3 and 4). Modeling studies indicate that previously highly endemic areas should aim for less residual infection than precontrol low-endemic areas [37]. Exact thresholds need to be determined [38, 39], in particular for infective blackflies and the new serologically based exposure assessment tools recommended by the WHO [40].

### Feasibility of Elimination by 2025

We chose to model MDA only until 2025 as targets were set for this year [13]. Our predictions suggest that this may not always be achievable, or could require intensification of intervention efforts. Hence, elimination targets may need adjustment to make them more attainable. Most problematic are areas with high precontrol endemicity and those with no or only short histories of control. Whereas most areas where control has not yet started are low endemic [15], there are some highly endemic areas where interventions began only recently (eg, Democratic Republic of Congo, Central African Republic, and South Sudan) [41]. We did not include areas with very high community microfilarial loads associated with a microfilarial prevalence exceeding 80% or even 90%, as the models are challenged by such extreme situations.

We did not investigate feasibility of elimination in areas low endemic for onchocerciasis and coendemic for loiasis, where current MDA strategies cannot be used, because of an unacceptably high risk of serious adverse events. This is a problem for many countries in the Central African region, including Angola, Cameroon, Central African Republic, Chad, Congo, Democratic Republic of Congo, Equatorial Guinea, Gabon, Nigeria, and South Sudan [42, 43]. Millions of people in these areas are therefore left untreated, presenting a source for reintroduction of onchocerciasis in previously eliminated areas. Alternative treatment strategies are urgently needed, such as the test-and-not-treat strategy [44]. This strategy seems to have similar impact as regular MDA when the same coverage can be achieved, which makes our results more generalizable. Modeling results for strategies in these areas will be presented elsewhere.

Alternative treatment strategies required to eliminate onchocerciasis have been identified by APOC [15, 45]. These include new drugs suitable for MDA, such as moxidectin [45, 46], which could significantly accelerate progress toward elimination, as our earlier modeling suggests [23]. Antibiotics targeting the *Wolbachia* endosymbionts of *O. volvulus* could be used within test-and-not-treat strategies [44], particularly in loaisis-coendemic areas because they are safe for coinfected patients [47]. Vaccines could have a substantial impact in a range of endemicity settings, and could markedly reduce host microfilarial loads in children and adolescents [24].

## CONCLUSIONS

It will be challenging to achieve onchocerciasis elimination targets in Africa and, in some areas, the target date should be shifted to be more attainable. Onchocerciasis control and elimination programs should aim for a high therapeutic coverage. Biannual treatment will be needed in hyperendemic settings with few years of past MDA. In the most highly endemic areas, even quarterly MDA with ≥80% coverage, if feasible, may not achieve elimination by 2025. The feasibility of localized VC by ground-based larviciding should be considered as an adjunct to MDA in some settings [34].

## Supplementary Data

Supplementary materials are available at *Clinical Infectious Diseases* online. Consisting of data provided by the authors to benefit the reader, the posted materials are not copyedited and are the sole responsibility of the authors, so questions or comments should be addressed to the corresponding author.

Supplementary Part 1

Supplementary Part 2

Supplementary Part 3

## Notes

***Author contributions.*** S. V. and M. W. contributed equally as first authors. M.-G. B. and W. A. S. contributed equally as last and senior authors.

***Acknowledgments.*** We are very grateful to Alex van der Steen for running the initial ONCHOSIM models and Anneke de Vos for developing the graphs, using R-script. Hans Remme gave very useful comments on an earlier version of this manuscript.

***Code availability.*** The models, along with a detailed description and program code, have been made available in a previous joint publication [22, 37].

***Disclaimer.*** The views, opinions, assumptions, or any other information set out in this article are solely those of the authors and should not be attributed to the funders, any person connected with the funders, or WHO.

***Financial support.*** This work was supported by the Bill & Melinda Gates Foundation in partnership with the Task Force for Global Health through the neglected tropical disease (NTD) Modelling Consortium (grant number OPP1053230).

***Supplement sponsorship.*** This article appears as part of the supplement “Reaching the 2020 Goals for Nine Neglected Tropical Diseases,” sponsored by the NTD Modelling Consortium.

***Potential conflicts of interest.*** All authors: No reported conflicts of interest. All authors have submitted the ICMJE Form for Disclosure of Potential Conflicts of Interest. Conflicts that the editors consider relevant to the content of the manuscript have been disclosed.
