# Supplementary material for: How Can Onchocerciasis Elimination in Africa Be Accelerated? Modeling the Impact of Increased Ivermectin Treatment Frequency and Complementary Vector Control
Source: Clin Infect Dis. 2018 Jun 1;66(Suppl 4):S267–74. doi: 10.1093/cid/cix1137 (PMC5982715; doi:10.1093/cid/cix1137)
Supplement: Supplementary Part 1 [file cix1137_suppl_supplementary_part_1.docx]

**How can onchocerciasis elimination in Africa be accelerated? Modelling the impact of increased ivermectin treatment frequency and complementary vector control**

Suzanne Verver, Martin Walker, Young Eun Kim, Grace Fobi, Afework H. Tekle, Honorat, G.M. Zouré, Samuel Wanji, Daniel A. Boakye, Annette C. Kuesel, Sake J. de Vlas, Michel Boussinesq, Maria-Gloria Basáñez, Wilma A. Stolk

**Online Supplement 1 (revision December 2017)**

Table S1. Five different pre-control endemicity levels and corresponding characteristics

| **Microfilarial prevalence in population aged ≥5 years** | **Annual biting rate** | **CMFL** | **microfilarial prevalence in total population (all ages)** |
| --- | --- | --- | --- |
| **ONCHOSIM** |  |  |  |
| 40% | 9,162 | 3.1 | 33.1% |
| 50% | 9,471 | 6.1 | 41.3% |
| 60% | 9,995 | 9.6 | 49.4% |
| 70% | 11,168 | 16.5 | 57.5% |
| 80% | 13,790 | 31.8 | 65.8% |
| **EPIONCHO** |  |  |  |
| 40% | 663 | 1.6 | 34.6% |
| 50% | 787 | 2.8 | 44.2% |
| 60% | 1,220 | 5.5 | 54.2% |
| 70% | 5,000 | 12.9 | 64.7% |
| 80% | 25,500 | 35.3 | 75.0% |

Mf = microfilaria

CMFL = community microfilarial load = geometric mean number of microfilariae per skin snip in population aged ≥ 20 years. For EPI-ONCHO CMFLs are approximations calculated by Monte Carlo simulation assuming all fertile females are mated assuming skin snip weighs 2 g.

**Table S2. ONCHOSIM quantification used to simulate onchocerciasis transmission.**

(this table has minor revisions compared to similar table in supplement of Walker & Stolk 2017)

| **Parameter** | **Value** | | **Source** |
| --- | --- | --- | --- |
| **Human demography** | | | |
| *Cumulative survival F*(*a*), by *age* | | | United Nations, 2013 |
| 0 | 1.000 | |  |
| 5 | 0.804 | |  |
| 10 | 0.772 | |  |
| 15 | 0.760 | |  |
| 20 | 0.740 | |  |
| 30 | 0.686 | |  |
| 50 | 0.509 | |  |
| 90 | 0.000 | |  |
| *Fertility rate per woman R*(*a*)*, by age* | | | United Nations, 2013 |
| 0–14 | 0.000 | |  |
| 15–19 | 0.109 | |  |
| 20–29 | 0.300 | |  |
| 30-49 | 0.119 | |  |
| 50+ | 0.000 | |  |
| Population trimming | 10% if population size exceeds 440. | | Assumption |
| **Transmission of infection** | | | |
| *General transmission parameters* |  | |  |
| Relative biting rate (*rbr*) | Multiplied with the reference *mbr* values, to modify the monthly and annual biting rate: varied between simulations. | |  |
| Seasonal variation in contribution to reservoir (*mbr*) | Reference *mbr* values (Jan-Dec):  We used mbr 2670, 2350, 1500, 1920, 1940, 1690, 2630, 3410, 3010, 3290, 3750, 2690 | | Alley 1994 & Entomological data collated by OCP |
| Transmission probability (*v*), i.e. the probability that an infective particle in the reservoir successfully develops into a parasite life stage that is capable of infecting a human host | *v* = 0.07345; see reference for the derivation of this value, given parameters for fly biology and development of infective L3 larvae within the fly. | | Coffeng et al., 2014 |
| Success ratio (*sr*) | *sr* = 0.0031 | | Plaisier, 1996; Duke, 1993 |
| Zoophily (*z, 1 -h*) | *z* = 0.04 ; *h* = 0.96 | | Habbema et al., 1996; expert opinion (OCP entomologists) |
| *Individual relative exposure to flies* |  | |  |
| Relative exposure by age and sex (*Exa*) | Zero at birth, linearly increasing between ages 0–20 from 0 to 1.0 for men and from 0 to 0.7 for women, and then constant from the age of 20 years onwards | | Plaisier, 1996 |
| Variation due to personal factors (fixed through life) given age and sex (*α_Exi_*) | Gamma distribution with mean 1.0 and shape and rate equal to 3.5 | | Plaisier, 1996; unpublished data from OCP |
| *Individual relative contribution to infection in the fly population* | | | |
| Variation by age and sex (*Coa*) | *Coa = Exa;* individual contribution and exposure to the cloud are perfectly correlated, given they are governed by the same fly bites. | | Assumption |
| Variation due to personal factors (fixed through life) given age and sex (*α_Coi_*) | *Coi = Exi*; individual contribution and exposure to the cloud are perfectly correlated, given they are governed by the same fly bites. | | Assumption |
| *Host immunity to incoming infections* | | |  |
| Average impact of host immunity (*α_Imm_*) | Assumed irrelevant for onchocerciasis, hence *α_Imm_ =* 0; i.e. no effect of immunity on incoming infections. | | Assumption |
| Immunological memory (*β_Imm_*) | Irrelevant given that *α_Imm_ =* 0. | | Assumption |
| **Life history and productivity of the parasite in the human host** | | | |
| Average worm lifespan (*Tl*) | 10 years | | Plaisier et al., 1991 |
| Variation in worm lifespan | Weibull distribution with shape 3.8. | | Assumption; Plaisier et al., 1991 |
| Prepatent period (*pp*) | 1 year | | Plaisier et al., 1991 which refers to Duke, 1980 and Prost, 1980 |
| Age-dependent microfilaria production capacity, *R(a)* | *R(a)* = 1 for 0 ≤ *a* < 5 | | Plaisier et al., 1991 which refers to Albeiz, 1985 and Karam et al., 1987 |
|  | *R(a)* = 1-((a-5)/15) for 5 ≤ *a* < 20 | |  |
|  | *R(a)* = 0 for *a* > 20 | |  |
|  |  | |  |
| Longevity of microfilariae within host (*Tm*) | 9 months | | Plaisier, 1996 |
| Mating cycle (*rc*) | 3 months | | Plaisier, 1991 and 1996 which refers to Schulz-Key and Karam, 1986 and Schulz-Key, 1990 |
| Male potential (*pot*) | 100 female worms. | | Plaisier, 1996 |
| *Density-dependent female worm reproductive capacity* |  | |  |
| Worm contribution to host load of infective material (*0*(.)) | 7.6 mf/worm | | Plaisier, 1996 |
| Exponential saturation of individual female worm productivity per worm present in host (*λ_z_*) | *λ_z_* = 0 i.e. no exponential saturation. | | Assumption |
| **Morbidity** | | | |
| Disease threshold (*Elc*) for blindness | Weibull distribution with mean 10.000 and shape 2.0 | | Coffeng et al., 2013 |
| Reduction in remaining life expectancy due to blindness (*rl*) | 50% | | Coffeng et al., 2013 which refers to partly published data from OCP; Dadzie et al., 1986;  Plaisier et al., 1990 which refers to Prost and Vaugelade 1981 and Kirkwood et al., 1983 |
| **Infection dynamics in the cloud** | | | |
| Cloud uptake of infectious material (*U*(.)) | Exponential saturating function with parameters *a* = 1.2, *b* = 0.0213, and *c* = 0.0861. | | Plaisier et al., 1991 (b) which refers to WHO, 1989 and Philippon, 1977. |
| Monthly cumulative survival of infective material in the central reservoir (*ψ*) | 0%; i.e. the cloud represents a cloud of vectors that transmit infection within the same month. | | Assumption |
| **Mass treatment coverage** | | | |
| Timing and coverage (*C_w_*_­_) | Varied between scenarios | |  |
| *Relative compliance* (*c_r_*(*k*, *s*)) *by age and sex* | | | Based on unpublished OCP data |
| age-group | cr(k,males) | cr(k,females) |  |
| 0-4 | 0 | 0 |  |
| 5-9 | 0.75 | 0.75 |  |
| 10-14 | 0.8 | 0.7 |  |
| 15-19 | 0.8 | 0.74 |  |
| 20-29 | 0.7 | 0.65 |  |
| 30-49 | 0.75 | 0.7 |  |
| 50+ | 0.8 | 0.75 |  |
| **Drug treatment** | | | |
| Proportion of microfilariae cleared from host | 100% | | Plaisier et al., 1995 |
| Duration of temporary reduction in female reproductive capacity (*Tr_0_*), average | 11 months | | Plaisier et al., 1995 |
| Permanent reduction in female worm reproductive capacity (*d*_0_), average | 34.9% | | Plaisier et al., 1995 |
| Proportion of adult worms killed (*m*_0_) | 0% | | Plaisier et al., 1995 |
| Relative effectiveness (*v*) | Weibull distribution with mean 1 and shape 2 | | Plaisier et al., 1995 |
| **Vector control** | | | |
| Timing | Not used. | |  |
| Coverage | Not used. | |  |
| **Surveys** |  | |  |
| Timing | Surveys are done at yearly intervals from 1988-2076. They are always done in month 6, i.e. exactly 12 or 6 months after annual or biannual treatment respectively. The simulation allows for a 200-year warming-up period before the first survey in 1998. | |  |
| Dispersal factor for worm contribution to measured density of infective material (*d*) | Exponential distribution with mean 1 | | Plaisier et al., 1991 |
| Variability in measured host load of infective material (here: mf per skin snip) | Poisson distribution with mean $ss\left( t \right)$ | | Plaisier et al., 1990 |

**References Table S2:**

Alley, E.S., Plaisier, A.P., Boatin, B.A., Dadzie, K.Y., Remme, J., Zerbo, G., Samba, E.M., 1994. The impact of five years of annual ivermectin treatment on skin microfilarial loads in the onchocerciasis focus of Asubende, Ghana. *Trans. R. Soc. Trop. Med. Hyg.* 88, 581-584. <http://dx.doi.org/10.1016/0035-9203(94)90172-4>.

Coffeng, L.E., Stolk, W.A., Zouré, H.G.M., Veerman, J.L., Agblewonu, K.B., Murdoch, M.E., Noma, M., Fobi, G., Richardus, J.H., Bundy, D.A.P., Habbema, D., de Vlas, S.J., Amazigo, U.V., 2013. African Programme for Onchocerciasis Control 1995-2015: model-estimated health impact and cost. *PLoS Negl. Trop. Dis*. 7, e2032. <http://dx.doi.org/10.1371/journal.pntd.0002032>.

Coffeng, L.E., Stolk, W.A., Hoerauf, A., Habbema, D., Bakker, R., Hopkins, A.D., de Vlas, S.J., 2014. Elimination of African onchocerciasis: modelling the impact of increasing the frequency of ivermectin mass treatment. *PLoS ONE* 9, e115886. <http://dx.doi.org/doi:10.1371/journal.pone.0115886>.

Dadzie KY, Remme J, Rolland A, Thylefors B.,1986. The effect of 7-8 years of vector control on the evolution of ocular onchocerciasis in West African savanna. *Trop Med Parasitol.* 37(3):263-70.

Duke, B.O.L., 1980. Observations on *Onchocerca volvulus* in experimentally infected chimpanzees. *Tropenmed.* *Parasitol.* 31, 41–54.

Duke, B.O.L., 1993. The population dynamics of *Onchocerca volvulus* in the human host. *Trop. Med. Parasitol.* 44**,** 61–68.

Habbema, J.D.F., van Oortmarssen, G.J., Plaisier, A.P., 1996. The ONCHOSIM model and its use in decision support for river blindness control. In Isham V, Medley G (Eds.), Models for infectious human diseases. Their structure and relation to data. Publications of the Newton Institute. Cambridge University Press, Cambridge, UK, pp. 360–380.

Karam, M., Schulz-Key, H., Remme, J., 1987. Population dynamics of *Onchocerca volvulus* after 7 to 8 years of vector control in West Africa. *Acta Trop*. 44, 445–457.

Kirkwood, B., Smith, P., Marshall, T., Prost, A., 1983. Relationships between mortality, visual acuity and microfilarial load in the area of the Onchocerciasis Control Programme. *Trans. R. Soc. Trop. Med. Hyg.* 77, 862–868.

Philippon, B., 1977. Etude de la transmission d' *Onchocerca volvulus* (Leuckart, 1983) Nematoda, Onchocercidae) par *Simulium damnosum* (Theobald, 1903) (Diptera, Simuliidae) en Afrique tropicale. *Travaux et Documents ORSTOM*, 63.

Plaisier, A.P., van Oortmarssen, G.J., Habbema, J.D., Remme, J., Alley, E.S., 1990. ONCHOSIM: a model and computer simulation program for the transmission and control of onchocerciasis. *Comput. Methods Programs Biomed.* 31, 43-56.

Plaisier, A.P., van Oortmarssen, G.J., Habbema, J.D., Remme, J., 1991. The reproductive lifespan of *Onchocerca volvulus* in West African savanna. *Acta Trop.* 48, 271-284.

Plaisier, A.P., van Oortmarssen G.J., Remme J., Alley E.S., Habbema J.D., 1991 (b). The risk and dynamics of onchocerciasis recrudescence after cessation of vector control. *Bull World Health Organ.* **69**(2): p. 169-78.

Plaisier, A.P., Soumbey-Alley, E., Boatin, B.A., van Oortmarssen, G.J., Remme, H., de Vlas, S.J., Bonneux, L., Habbema, D.F., 1995. Irreversible effects of ivermectin on adult parasites in onchocerciasis patients in the Onchocerciasis Control Programme in West Africa. *J. Infect. Dis.* 172, 204-210. <http://dx.doi.org/10.1093/infdis/172.1.204>.

Plaisier, A.P., 1996. Modelling onchocerciasis transmission and control. PhD thesis, Erasmus University, Rotterdam, The Netherlands. Available <http://repub.eur.nl/pub/21404/>.

Prost, A., 1980. Latence parasitaire dans I'oncbocereose. *Bull Wld. Hlth. Organ.* 58, 923-925.

Prost, A., Vaugelade, J., 1981. La surmortalité des aveugles en zone de savane ouest-africaine. *Bull. World Health Organ.* 59, 773-776.

Schulz-Key, H., Karam, M., 1986. Periodic reproduction of *Onchocerca volvulus*. *Parasitol. Today* 2, 284–286.

Schulz-Key, H., 1990. Observations on the reproductive biology of *Onchocerca volvulus*. *Acta Leiden*. 59, 27–43.

United Nations Department of Economic and Social Affairs Population Division, *World Population* *Prospects: the 2012 revision, Volume I: Comprehensive Tables*, 20.

World Health Organization, 1989. Onchocerciasis Control Programme in West Africa: report of the annual OCP research meeting, 20-24 March 1989. (unpublished).

**Table S3. EPIONCHO parameter values and definitions**

## **Table S3a** Coverage and adherence to mass treatment.

| **Parameter** | **Definition** | **Value** | **Reference** |
| --- | --- | --- | --- |
| *ρ_F_*=*ρ_M_* | proportion of human women (set equal to *ρ_M_*) | 0.5 | Filipe et al., 2005 |
| *ρ_d_*_=1_ | proportion of human hosts eligible for treatment with ivermectin (aged ≥ 5 years) treated every round | Defined by therapeutic coverage and systematic non-adherence | Walker et al., 2017 |
| *ρ_d_*_=2_ | proportion of human hosts eligible for treatment with ivermectin (aged ≥ 5 years) treated every other round | Defined by therapeutic coverage and systematic non-adherence | Walker et al., 2017 |
| *ρ_d_*_=3_ | proportion of human hosts systematically non-adherent | 0.05 | Walker et al., 2017 |

## **Table S3b** Human host demographic structure*.*

| Parameter / function | Definition | Value | Reference |
| --- | --- | --- | --- |
| *ρ*(*a*) =*μ_H_*exp(-*μ_H_a*)/[1 – exp(–*μ_H_a_max_*)] | truncated exponential distribution of proportion of human hosts of age *a* | NA | Filipe et al., 2005 |
| *a_max_* | maximum age of human hosts | 80 years | Filipe et al., 2005 |
| *μ_H_* | human host population distribution inverse scale parameter | 0.04 | Filipe et al., 2005 |

## **Table S3c** Human host exposure to blackfly bites*.*

| Parameter / function | Definition | Value | Reference |
| --- | --- | --- | --- |
| Ω*_s_*(*a*) = *E_s_γ_s_E*_0_ for *a* < *q*  *= E_s_γ_s_*exp[-*α_s_*(*a* – *q*)] | age- and sex-specific exposure to biting blackfly vectors, including normalization constant *γ_s_* | NA | Filipe et al., 2005 |
| *E*_0_ | relative exposure to blackfly bites at birth relative to that at age *q* | 0.1 | Filipe et al., 2005 |
| Q = *E_M_*/*E_F_* | relative men to women exposure to biting blackfly vectors (here set to 1) | 1.0 | Walker et al., 2017 |
| *α_F_ = α_M_* | rate of change in exposure to blackly vectors with age among women (here set equal to *α_M_*) | 0.0058 | Walker et al., 2017; Filipe et al., 2005 |
| *α_M_ = α_F_* | rate of change in exposure to blackly vectors with age among men (here set equal to *α_F_*) | 0.0058 | Walker et al., 2017; Filipe et al., 2005 |
| *q* | period of initial increase in exposure to vector bites during childhood | 0.00 | Filipe et al., 2005 |

## **Table S3d** Parasite and vector demographic rates.

| Parameter / function | Definition | Value | Reference(s) |
| --- | --- | --- | --- |
| *μ_V_* | per capita mortality rate of blackfly vectors | 23 | Walker et al., 2017; Basáñez & Boussinesq, 1999 |
| *ε* | per capita rate of microfilarial production per female adult parasite within human host | 0.75 | Walker et al., 2017; Basáñez & Boussinesq, 1999 |
| *ε** = (*λ*_0_+*σ_W_*_0_+*ω*)/*ω* | per capita rate of microfilarial production per fertile female adult parasite within human host | Defined by *ε*, *λ*_0_, *σ_W_*_0_, and *ω* | Basáñez et al., 2008 |
| *σ_W_* = *mσ_W_*_0_ | per capita rate of progression of adult parasites through nominal age compartments | NA | Walker et al., 2017; |
| 1/*σ_W_*_0_ | life-expectancy of adult parasites within human hosts | 9.2 | Walker et al., 2017; Plaisier et al., 1991 |
| *m* = 2/*σ_W_*_0_ | number of nominal age compartments of adult parasites you used *m* in the text; *n* better | Defined by *σ_W_*_0_ | Walker et al., 2017; Plaisier et al., 1991 |
| *σ_M_* | per capita mortality rate of mf within human hosts | 0.68 | Walker et al., 2017; Basáñez & Boussinesq, 1999; Plaisier et al., 1995 |
| *σ_L_* | per capita mortality rate of L3 larvae within blackfly vectors | 62.5 | Walker et al., 2017; Basáñez & Boussinesq, 1999 |
| *ω* | per capita rate of progression from non-fertile to fertile adult parasites | 0.57 | Walker et al., 2017; Basáñez et al., 2008 |
| *λ*_0_ | per capita rate of reversion from fertile to non-fertile adult parasites | 0.31 | Walker et al., 2017; Basáñez et al., 2008 |
| *ν*_1_ | per capita rate of progression from L1 to L2 larvae within blackfly vectors | 73.7 | Walker et al., 2017; Eichner et al., 1991 |
| *ν*_2_ | per capita rate of progression from L2 to L3 larvae within blackfly vector | 135.9 | Walker et al., 2017; Eichner et al., 1991 |

Rates are per year; durations are in years; proportions are dimensionless.

## **Table S3e** Transmission rates and regulation of parasite population in human hosts

| Parameter / function | Definition | Value | Reference |
| --- | --- | --- | --- |
| ABR *= βV*/*H =* (*V*/*H*)(*h*/*g*) | annual biting rate of blackfly vectors | see  Table S1 | Basáñez & Boussinesq, 1999 |
| *V*/*H* = ABR(*g*/*h*) | vector to host ratio | NA | Basáñez & Boussinesq, 1999 |
| Φ[*W_s_*_,_*_d,j_*(*t*,*a*)] =  1+[1−*W_s_*_,_*_d,j_*(*t*,*a*)/*k_W_*]^-(^*^kW+^*^1)^ | female worm mating probability | NA | May, 1977 |
| *k_W_ = k_W_*_0_ + *k_W_*_1_*W_s_*_,_*_d,j_*(*t*,*a*) | overdispersion of negative binomial distribution describing the distribution of adult parasites among human hosts | NA | Walker et al, 2017 |
| *k_W_*_0_ | 0.024 |  | Walker et al, 2017 |
| *k_W_*_1_ | ~ 0.0 |  | Walker et al, 2017 |
| *k_M_* = *k_M_*_0_exp[*k_M_*_1_*M_s_*(*t*,*a*)] | overdispersion of negative binomial distribution describing the distribution of mf in the skin of human hosts | NA | Walker et al, 2017; Bottomley et al., 2016 |
| *k_M_*_0_ | 0.43 |  | Walker et al, 2017 |
| *k_M1_* | 0.021 |  | Walker et al, 2017 |
| Π*_Η_*[*L3*(*t*)] =  [*δ*_H0_ + *δ*_H∞_*c*_H_*mβL3*(*t*)] /  [1 + *c*_H_*mβL3*(*t*)] | proportion of L3 larvae developing into adult worms within the human host, per bite | NA | Basáñez & Boussinesq, 1999; Basáñez et al., 2002 |
| *δ*_H0_ | proportion of L3 larvae developing to the adult stage within the human host, per bite, when *mβL3*(*t*) → 0 | 0.12 | Walker et al., 2017; Filipe et al., 2005; Basáñez et al., 2002 |
| *δ*_H∞_ | proportion of L3 larvae developing to the adult stage within the human host, per bite, when *mβL3*(*t*) → ∞ | 4.6x10^-3^ | Walker et al., 2017; Filipe et al., 2005; Basáñez et al., 2002 |
| *c*_H_ | severity of density-dependent limitation of parasite establishment within humans | 1.7x10^-3^ | Walker et al., 2017; Filipe et al., 2005; Basáñez et al., 2002 |

Rates are per year; durations are in years; proportions are dimensionless

## **Table S3f** Transmission rates and regulation of parasite population in blackfly vectors

| Parameter / function | Definition | Value | Reference |
| --- | --- | --- | --- |
| *h* | human blood index (fraction of blood meals taken on humans) | 0.67 | Walker et al., 2017; Lamberton et al., 2016 |
| 1/*g* | reciprocal of the length of the gonotrophic cycle | 101 | Walker et al., 2017; Basáñez & Boussinesq, 1999 |
| *a_H_* | proportion of L3 larvae shed per bite | 0.82 | Walker et al., 2017 ; Basáñez & Boussinesq, 1999 |
| Π*_V_*[*M_s_*(*t*,*a*)] =  *δ*_V0_exp[-*c_V_a_V_M_s_*(*t*,*a*)]^†^ | proportion of mf per mg developing into infective larvae within the blackfly vector host per bite | NA | Churcher et al., 2006 |
| *δ*_V0_ | proportion of mf per mg developing to the infective stage per bite when *M_s_*(*t*,*a*) → 0 | 0.017 | Walker et al., 2017 |
| *c_V_* | severity of density-dependent limitation of larval development per dermal microfilaria | 0.0045 | Walker et al., 2017 |
| *a_V_* | proportion of mf per mg of skin ingested per bite | 0.56 | Walker et al., 2017; Basáñez & Boussinesq, 1999 |
| *α_V_* | per capita excess rate mortality on blackfly vectors induced by mf | 0.44 | Walker et al., 2017; Basáñez & Boussinesq, 1999 |

Rates are per year; durations are in years; proportions are dimensionless.

## **Table S3g** Pharmacodynamics of ivermectin

| Parameter / function | | Definition | Value | Reference(s) |
| --- | --- | --- | --- | --- |
| *ψ_d,j_* =1 for *j* = 0  *ψ_d,j_* =(1 − ζ)*^j^*^−1^ for *j* >1 | modifying function of fertility of fertile female parasites exposed to *j* treatments | | NA | Turner et al., 2013; Basáñez et al., 2016 |
| ζ | cumulative reduction in female parasite fertility per exposure to treatment | | 0.35 | Plaisier et al., 1995 |
| *λ_j_*_1_(*τ*) = *λ*_1_^max^exp(-*φτ*) | treatment induced per capita rate of reversion from fertile to non-fertile adult parasites at time *τ* since the last treatment | | NA | Basáñez et al., 2008 |
| *λ*_1_^max^ | maximum rate of treatment-induced sterility | | 32.4 | Basáñez et al., 2008 |
| *φ* | rate of decay of treatment induced sterilisation | | 19.6 | Basáñez et al., 2008 |
| *σ_M_*_1,_*_d_*(*τ*) = (*τ + υ*)^-^*^κ^* | excess mortality rate of mf due to treatment | | ΝΑ | Basáñez et al., 2008 |
| *υ* | constant to allow for very large yet finite microfilaricidal effect at treatment | | 9.6×10^-3^ | Basáñez et al., 2008 |
| *κ* | shape parameter for excess mortality of mf following treatment | | 1.25 | Basáñez et al., 2008 |

Rates are per year; durations are in years; proportions are dimensionless.

# **References Table 3**

Basáñez, M.G., Boussinesq, M., 1999. Population biology of human onchocerciasis. *Philos. Trans. R. Soc. Lond. B Biol. Sci.* 354, 809-826. <http://dx.doi.org/10.1098/rstb.1999.0433>

Basáñez, M.G., Collins, R.C., Porter, C.H., Little, M.P., Brandling-Bennett, D., 2002. Transmission intensity and the patterns of *Onchocerca volvulus* infection in human communities. *Am. J. Trop. Med. Hyg.* 67, 669-679.

Basáñez, M.G., Pion, S.D.S., Boakes, E., Filipe, J.A.N., Churcher, T.S., Boussinesq, M., 2008. Effect of single dose ivermectin on *Onchocerca volvulus*: a systematic review and meta-analysis. *Lancet Infect. Dis.* 8, 310-22. d <http://dx.doi.org/10.1016/S1473-3099(08)70099-9>.

Basáñez, M.G., Walker, M., Turner, H.C., Coffeng, L.E., de Vlas, S.J., Stolk, W.A., 2016. River blindness: mathematical models for control and elimination. *Adv. Parasitol. Adv. Parasitol.* 94, 247-341. <http://dx.doi.org/10.1016/bs.apar.2016.08.003>.

Bottomley, C., Isham, V., Vivas-Martinez, S., Kuesel, A.C., Attah, S.K., Opoku, N.O., Lustigman, S., Walker, M., Basáñez, M.G., 2016. Modelling Neglected Tropical Diseases diagnostics: the sensitivity of skin snips for *Onchocerca volvulus* in near elimination and surveillance settings. *Parasit. Vectors* 9, 343. <http://dx.doi.org/10.1186/s13071-016-1605-3>.

Churcher, T.S., Filipe, J.A.N., Basáñez, M.G., 2006. Density dependence and the control of helminth parasites. *J. Anim. Ecol.* **75**, 1313-1320. <http://dx.doi.org/10.1111/j.1365-2656.2006.01154.x>.

Eichner, M., Renz, A., Wahl, G., Enyong, P., 1991. Development of *Onchocerca volvulus* microfilariae injected into *Simulium* species from Cameroon. *Met. Vet. Entomol.* 5, 293-297. <http://dx.doi.org/10.1111/j.1365-2915.1991.tb00555.x>.

Filipe, J.A.N., Boussinesq, M., Renz, A., Collins, R.C., Vivas-Martinez, S., Grillet, M.E., Little, M.P., Basáñez, M.G., 2005. Human infection patterns and heterogeneous exposure in river blindness. *Proc. Natl. Acad. Sci. U S A*. 102, 15265-15270. <http://dx.doi.org/10.1073/pnas.0502659102>.

Lamberton, P.H.L., Cheke, R.A., Walker, M., Winskill, P., Crainey, J.L., Boakye, D.A., Osei-Atweneboana, M.Y., Tirados, I., Wilson, M.D., Sampson, O., Post, R.J., Basáñez, M.G., 2016. Onchocerciasis transmission in Ghana: the human blood index of sibling species of the *Simulium damnosum* complex. *Parasit. Vectors* 9, 432. <http://dx.doi.org/10.1186/s13071-016-1703-2>.

May, R.M., 1977. Togetherness among schistosomes: its effects on the dynamics of the infection. *Math. Biosci.* 35, 301-343. <http://dx.doi.org/10.1016/0025-5564(77)90030-X>.

Plaisier, A.P., van Oortmarssen, G.J., Habbema, J.D., Remme, J., 1991. The reproductive lifespan of *Onchocerca volvulus* in West African savanna. 48, 271-284.

Plaisier, A.P., Soumbey-Alley, E., Boatin, B.A., van Oortmarssen, G.J., Remme, H., de Vlas, S.J., Bonneux, L., Habbema, D.F., 1995. Irreversible effects of ivermectin on adult parasites in onchocerciasis patients in the Onchocerciasis Control Programme in West Africa. *J. Infect. Dis.* 172, 204-210. <http://dx.doi.org/10.1093/infdis/172.1.204>.

Turner, H.C., Churcher, T.S., Walker, M., Osei-Atweneboana, M.Y., Prichard, R.K., Basáñez, M.G., 2013. Uncertainty surrounding projections of the long-term impact of ivermectin treatment on human onchocerciasis. PLoS *Negl. Trop. Dis.* 7, e2169. <http://dx.doi.org/10.1371/journal.pntd.0002169>.

Walker M, Stolk WA, Dixon MA, Bottomley C, Diawara L, Traoré MO, de Vlas SJ, Basáñez MG, 2017. Modelling the elimination of river blindness using long-term epidemiological and programmatic data from Mali and Senegal. *Epidemics*;18:4-15.
