# Supplementary material for: How Can Onchocerciasis Elimination in Africa Be Accelerated? Modeling the Impact of Increased Ivermectin Treatment Frequency and Complementary Vector Control
Source: Clin Infect Dis. 2018 Jun 1;66(Suppl 4):S267–74. doi: 10.1093/cid/cix1137 (PMC5982715; doi:10.1093/cid/cix1137)
Supplement: Supplementary Part 2 [file cix1137_suppl_supplementary_part_2.docx]

**How can onchocerciasis elimination in Africa be accelerated? Modelling the impact of increased ivermectin treatment frequency and complementary vector control**

Suzanne Verver, Martin Walker, Young Eun Kim, Grace Fobi, Afework H. Tekle, Honorat G.M. Zouré, Samuel Wanji, Daniel A. Boakye, Annette C. Kuesel, Sake J. de Vlas, Michel Boussinesq, Maria-Gloria Basáñez, Wilma A. Stolk

**Online Supplement 2: ONCHOSIM figures**

**Table of Contents**

[Introduction to the supplement 3](#_Toc491417120)

[Historic prevalence: 40%. 4](#_Toc491417121)

[Treatment naive. 4](#_Toc491417122)

[Past 5 years annual MDA. 5](#_Toc491417123)

[Past 10 years annual MDA. 6](#_Toc491417124)

[Past 15 years annual MDA. 7](#_Toc491417125)

[Past 20 years annual MDA. 8](#_Toc491417126)

[Past 5 years bi-annual MDA. 9](#_Toc491417127)

[Historic prevalence: 50%. 10](#_Toc491417128)

[Treatment naive. 10](#_Toc491417129)

[Past 5 years annual MDA. 11](#_Toc491417130)

[Past 10 years annual MDA. 12](#_Toc491417131)

[Past 15 years annual MDA. 13](#_Toc491417132)

[Past 20 years annual MDA. 14](#_Toc491417133)

[Past 5 years bi-annual MDA. 15](#_Toc491417134)

[Historic prevalence: 60%. 16](#_Toc491417135)

[Treatment naive. 16](#_Toc491417136)

[Past 5 years annual MDA. 17](#_Toc491417137)

[Past 10 years annual MDA. 18](#_Toc491417138)

[Past 15 years annual MDA. 19](#_Toc491417139)

[Past 20 years annual MDA. 20](#_Toc491417140)

[Past 5 years bi-annual MDA. 21](#_Toc491417141)

[Historic prevalence: 70%. 22](#_Toc491417142)

[Treatment naive. 22](#_Toc491417143)

[Past 5 years annual MDA. 23](#_Toc491417144)

[Past 10 years annual MDA. 24](#_Toc491417145)

[Past 15 years annual MDA. 25](#_Toc491417146)

[Past 20 years annual MDA. 26](#_Toc491417147)

[Past 5 years bi-annual MDA. 27](#_Toc491417148)

[Historic prevalence: 80%. 28](#_Toc491417149)

[Treatment naive. 28](#_Toc491417150)

[Past 5 years annual MDA. 29](#_Toc491417151)

[Past 10 years annual MDA. 30](#_Toc491417152)

[Past 15 years annual MDA. 31](#_Toc491417153)

[Past 20 years annual MDA. 32](#_Toc491417154)

[Past 5 years bi-annual MDA. 33](#_Toc491417155)

**Title of the figures: Estimated impact of intervention on onchocerciasis microfilaria prevalence in different settings before and after switch to a new control strategy in 2019.**

**Introduction to the supplement**

Each figure depicts the microfilarial prevalence dynamics until 2035 in individuals aged ≥ 5 years during simulated interventions against onchocerciasis in different endemicity settings before and after a switch to a different intervention strategy implemented from 2019 to 2025 (excepting vector control implemented for 15 years which continued until 2035). Each figure depicts the simulated dynamics in settings with a pre-control microfilarial prevalence of 40, 50, 60, 70 or 80% with different histories of past control and future scenarios of no mass drug administration (MDA), annual MDA for 5,10,15 or 20 years, biannual MDA for 5 years with or without 5 or 15 years of complementary vector control. The dynamics elicited by quarterly MDA are not shown to prevent crowding of the figures. For ONCHOSIM mf prevalences represent the mean prevalence per 1000 model runs.

# Historic prevalence: 40%.

## Treatment naive.


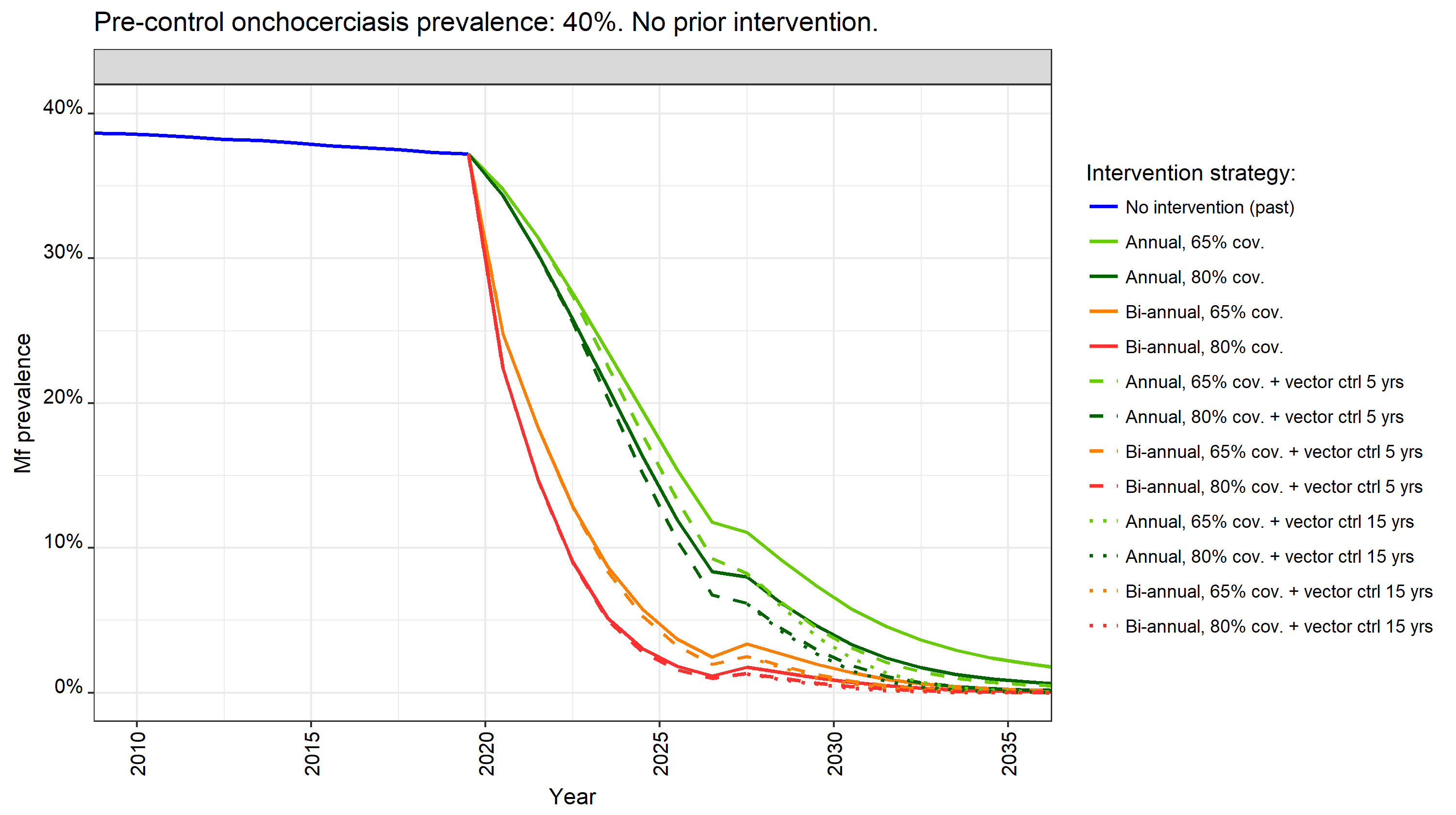

**Figure 1.1**

## Past 5 years annual MDA.


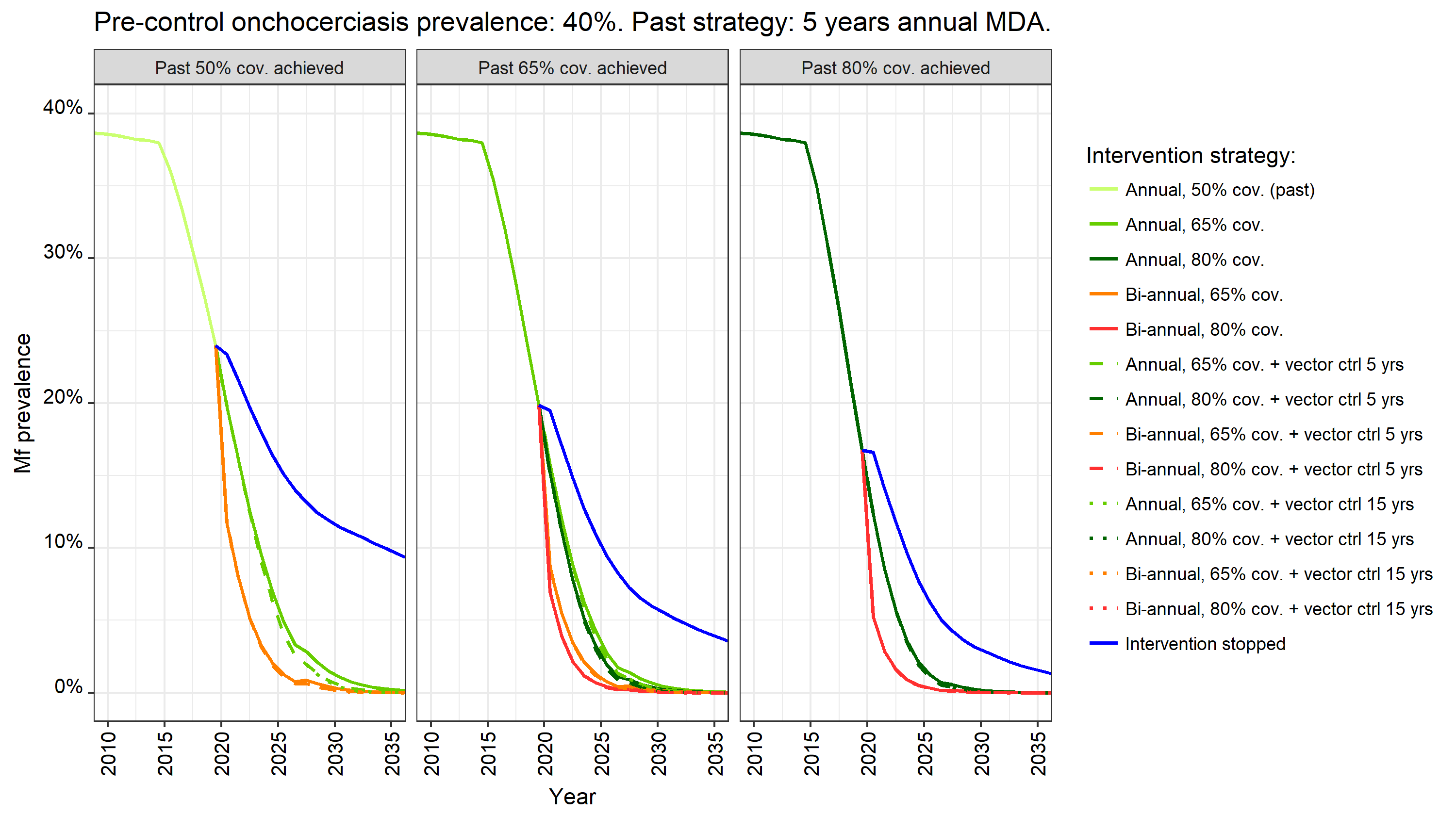

**Figure 1.2**

## Past 10 years annual MDA.


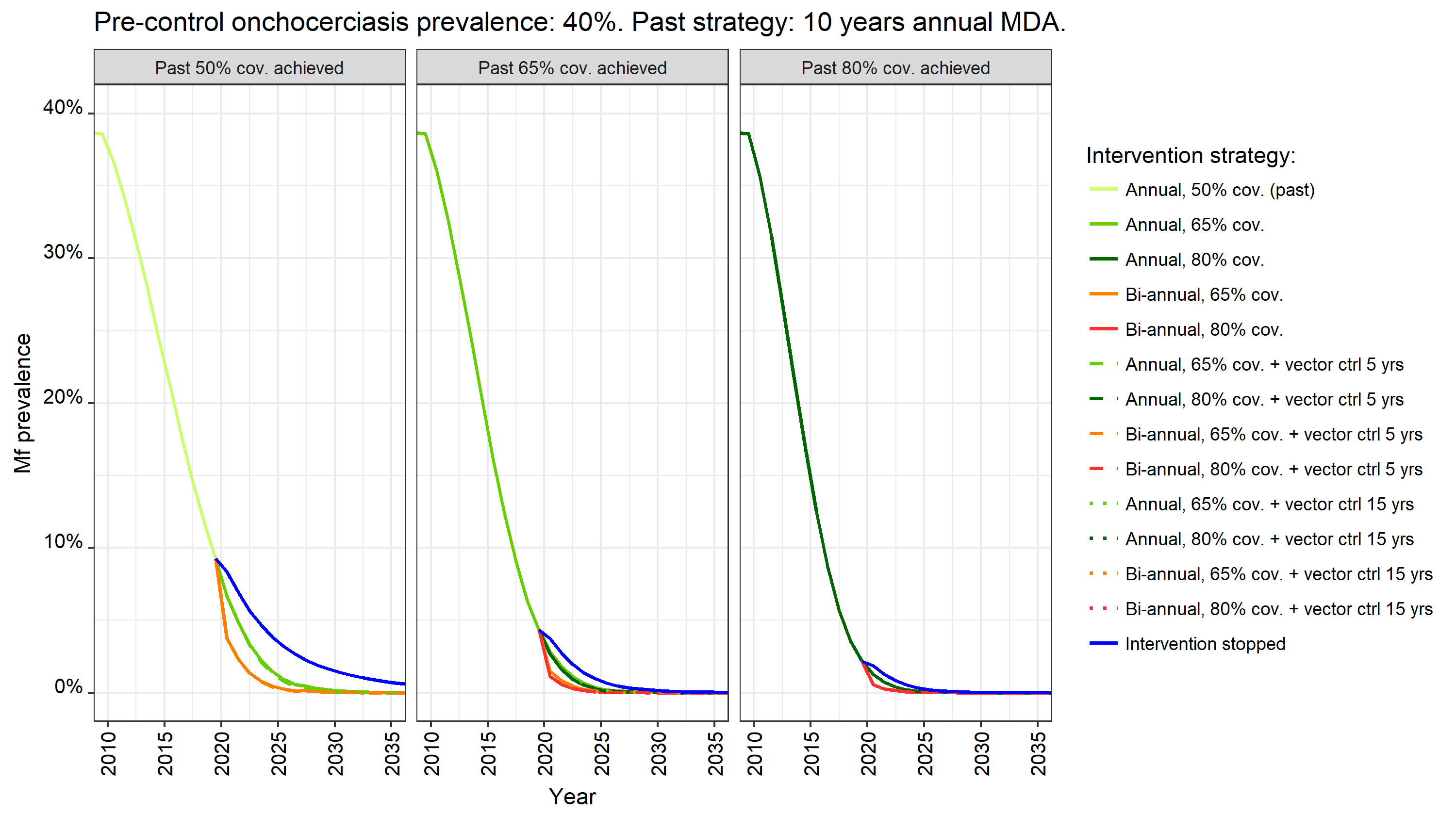

**Figure 1.3**

## Past 15 years annual MDA.


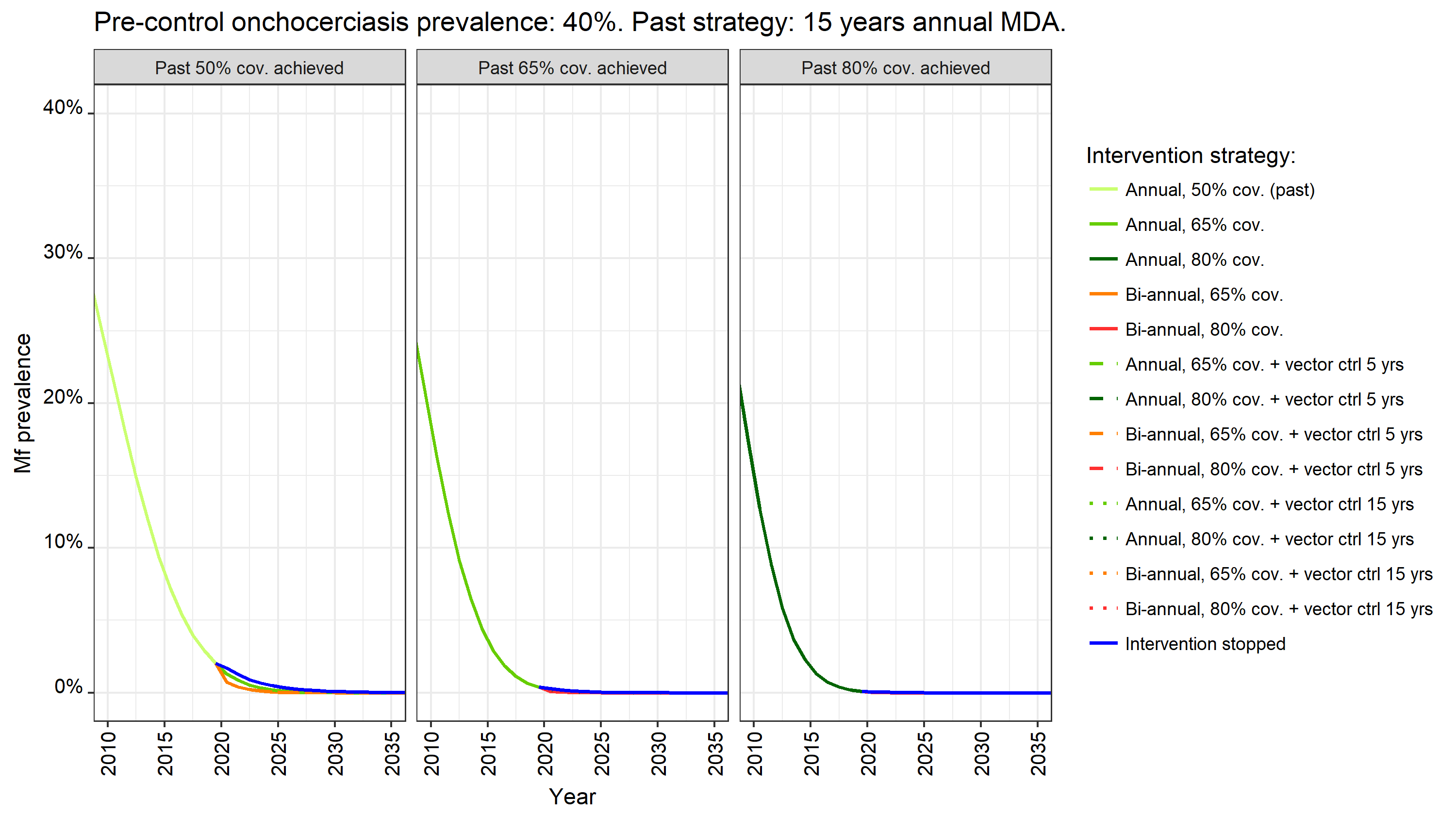

**Figure 1.4**

## Past 20 years annual MDA.


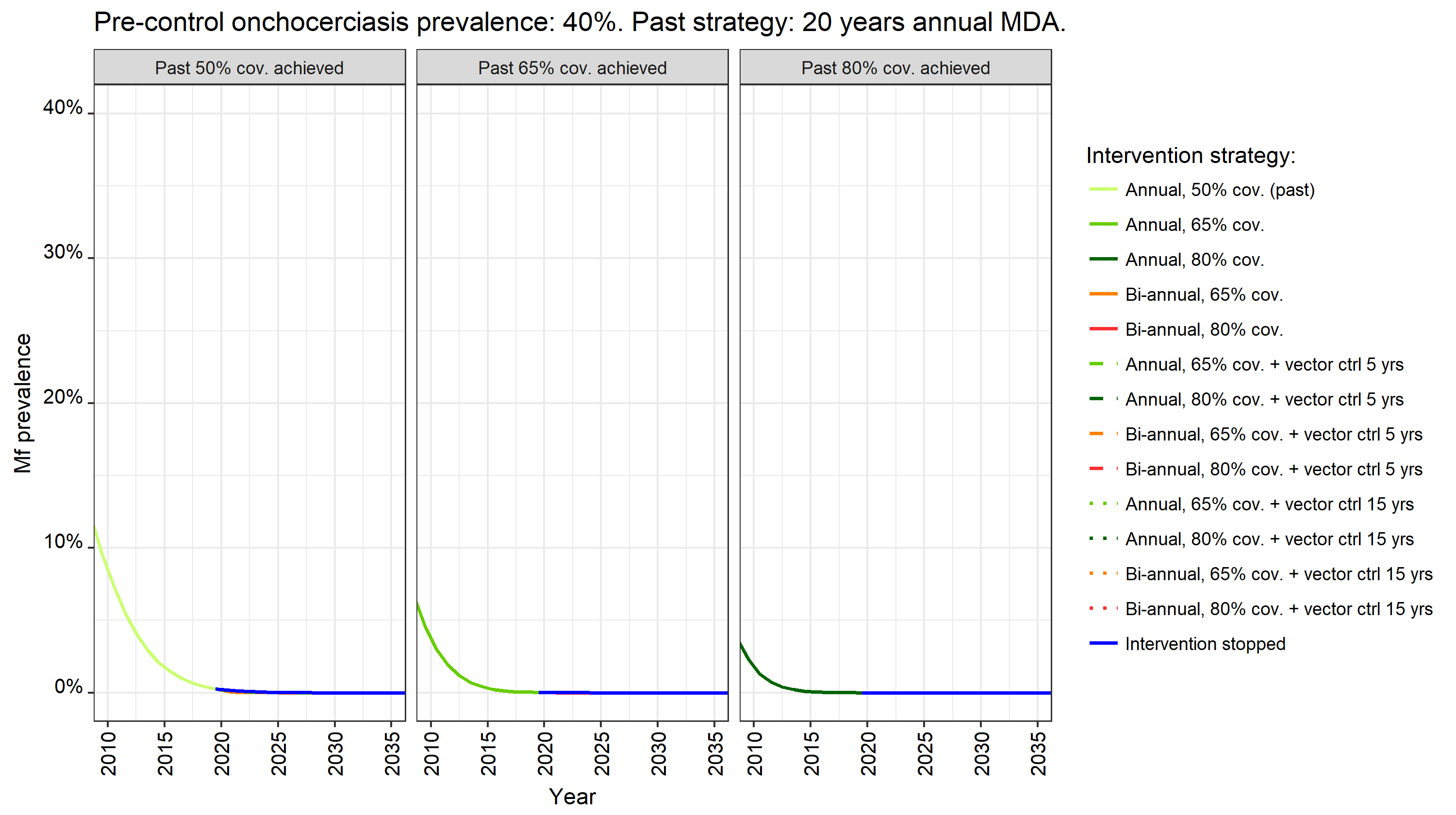

**Figure 1.5**

## Past 5 years bi-annual MDA.


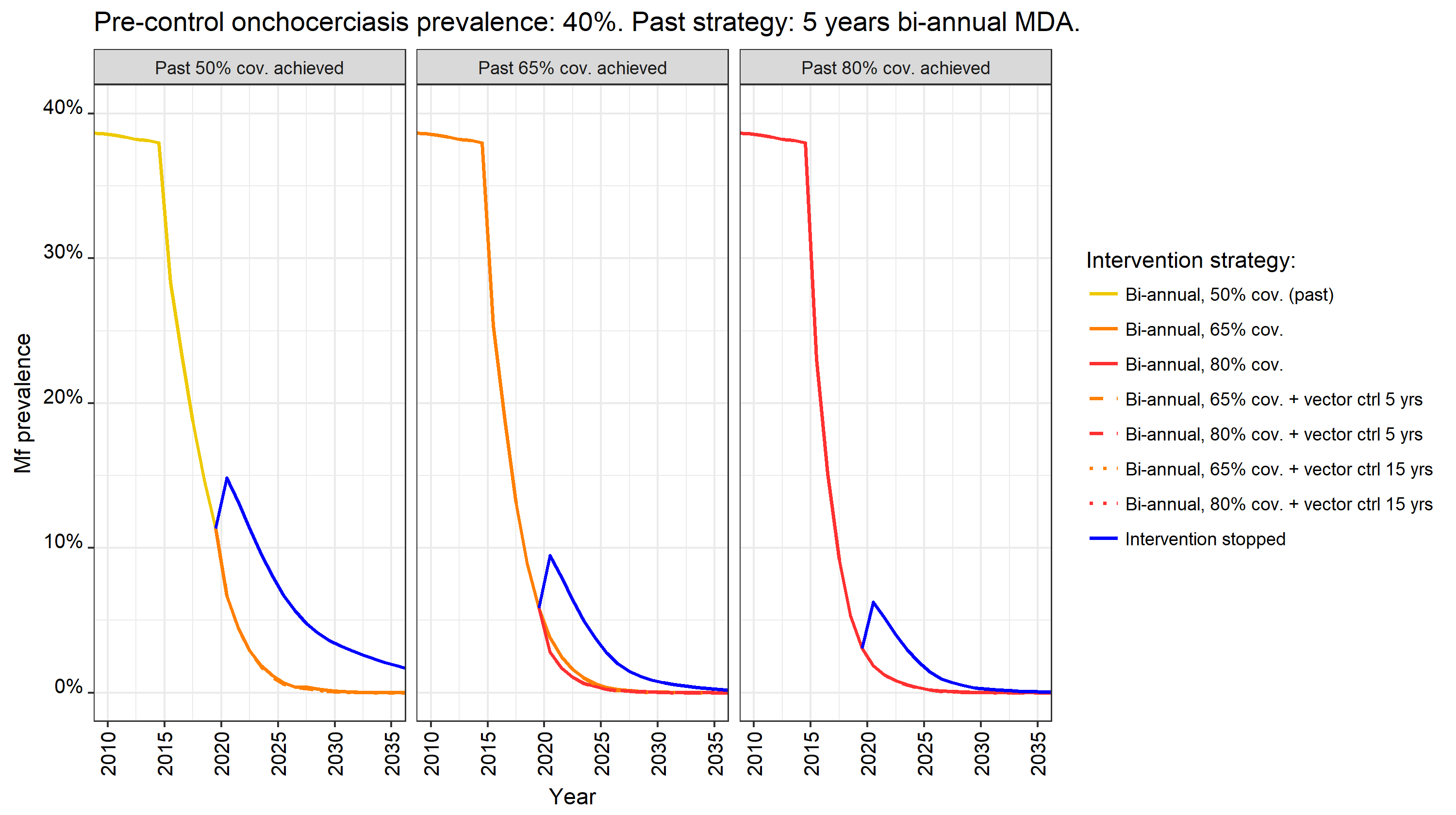

**Figure 1.6**

# Historic prevalence: 50%.

## Treatment naive.


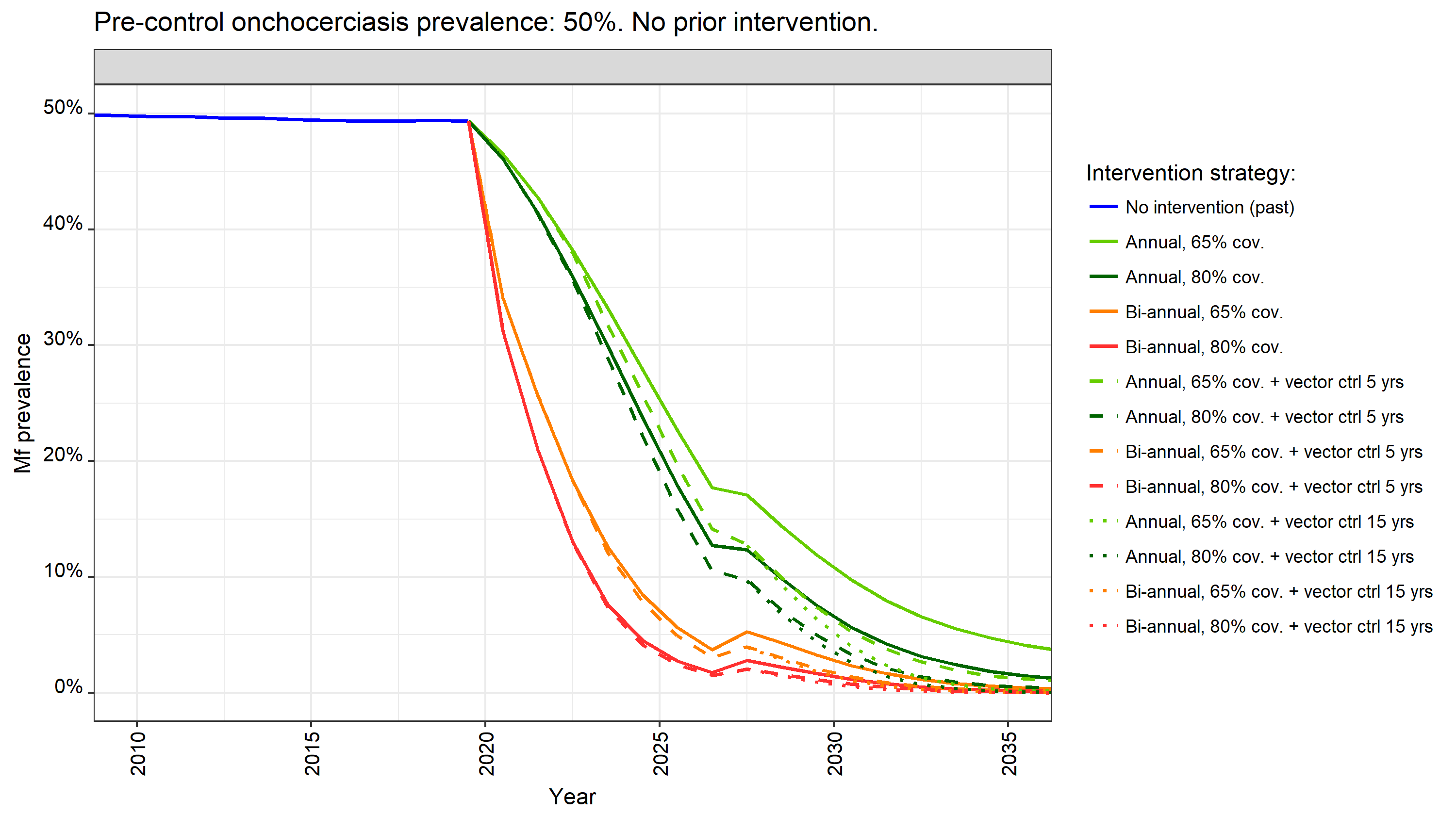

**Figure 2.1**

## Past 5 years annual MDA.


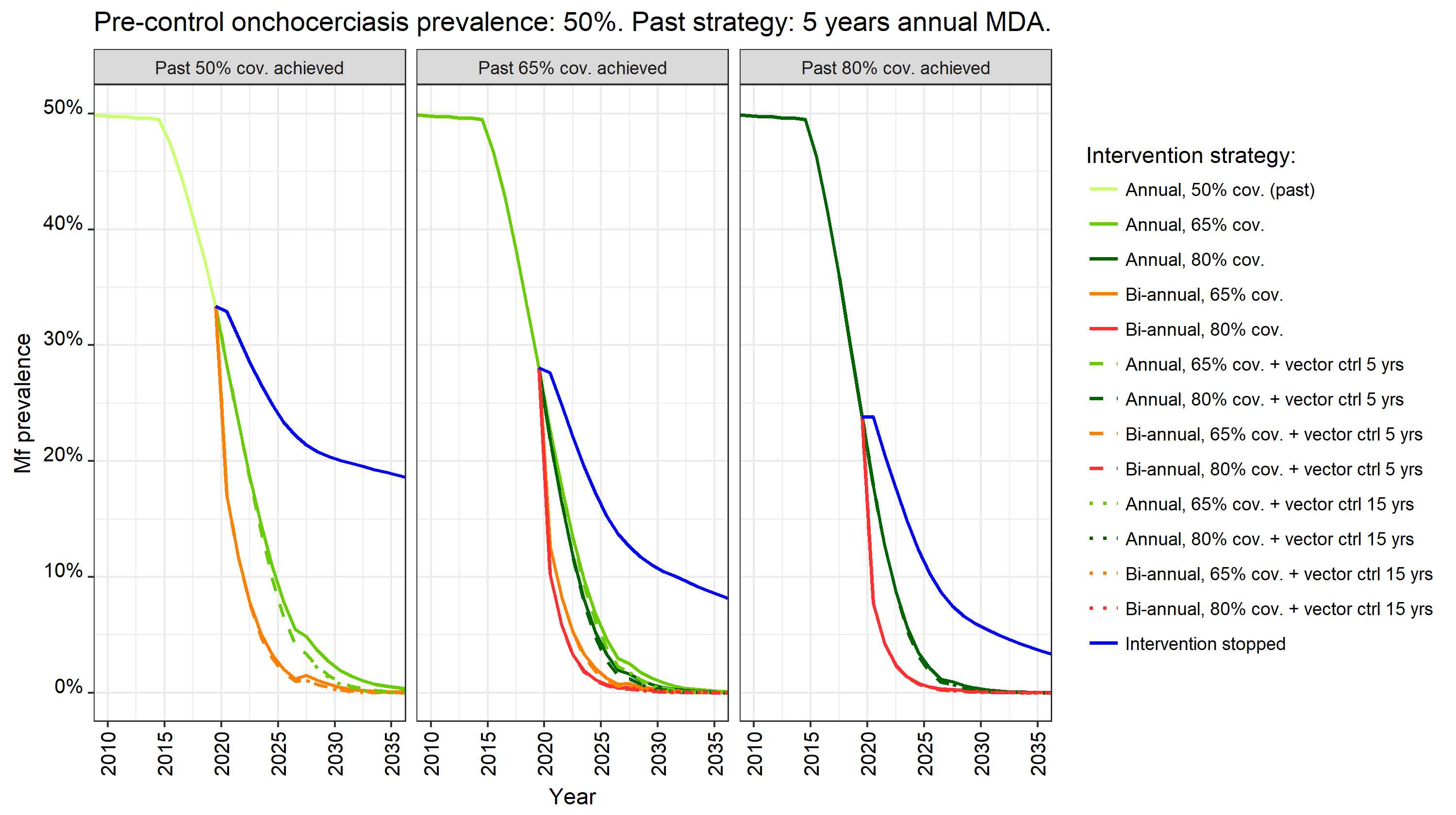

**Figure 2.2**

## Past 10 years annual MDA.


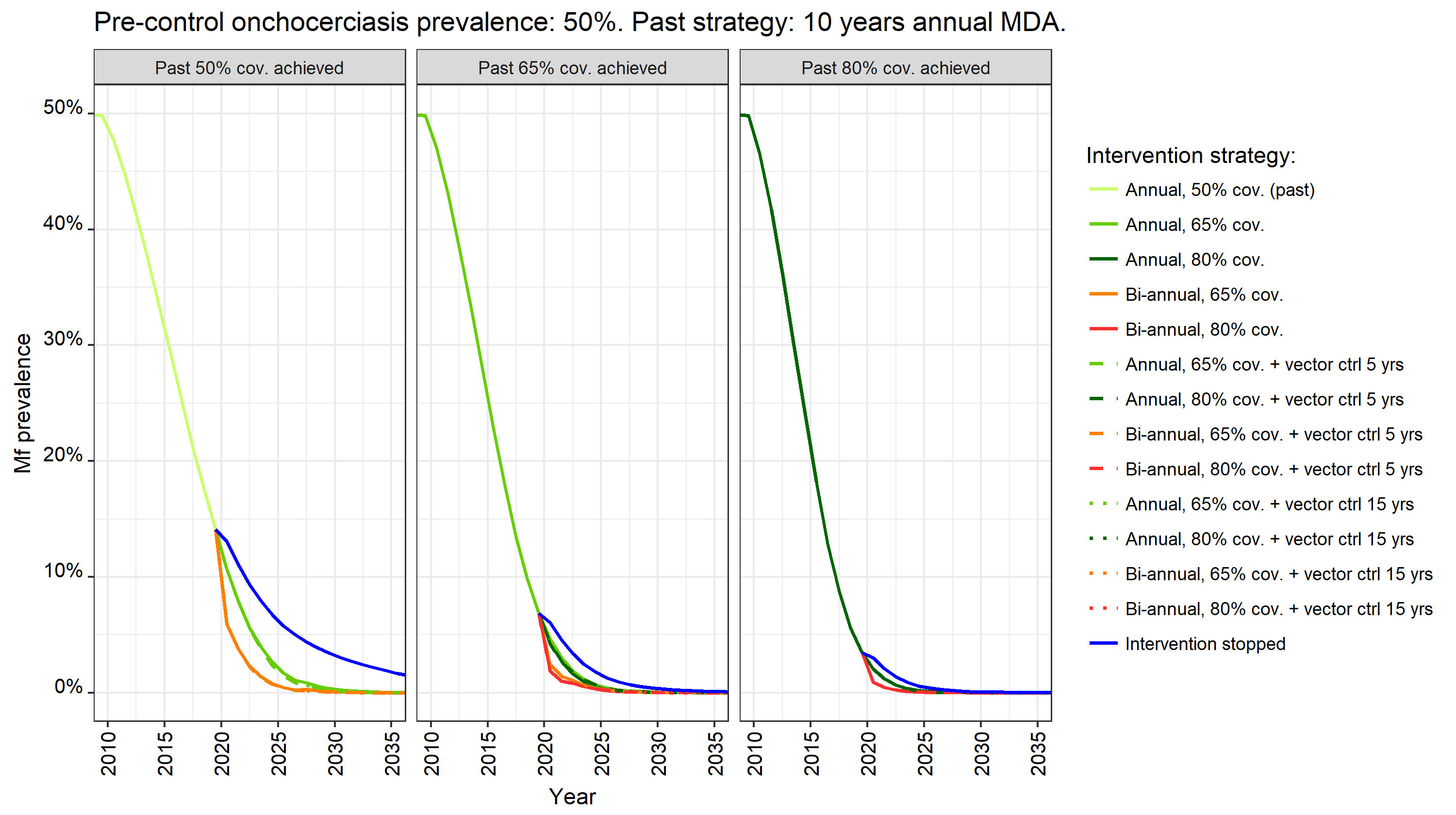

**Figure 2.3**

## Past 15 years annual MDA.


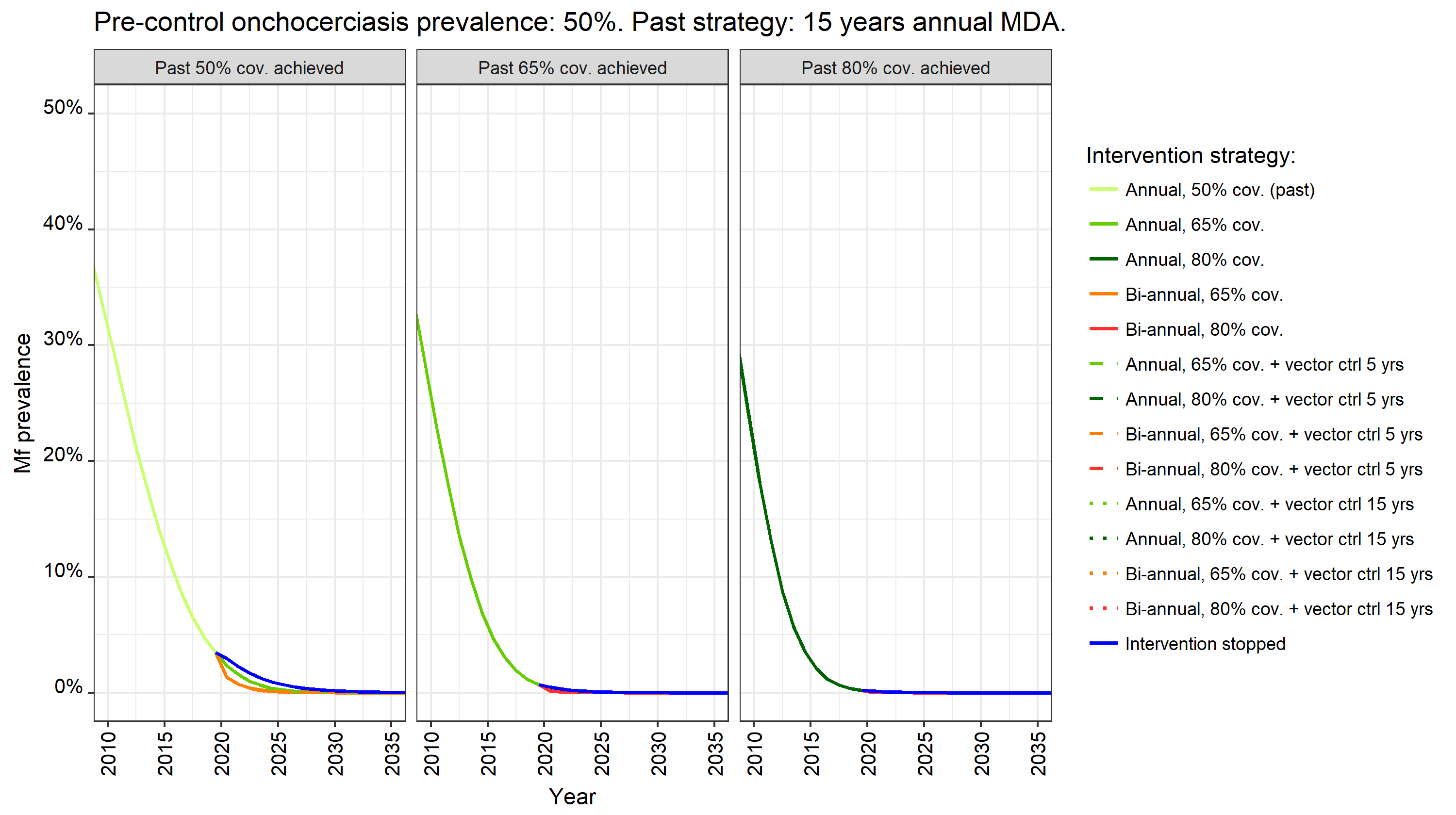

**Figure 2.4**

## Past 20 years annual MDA.


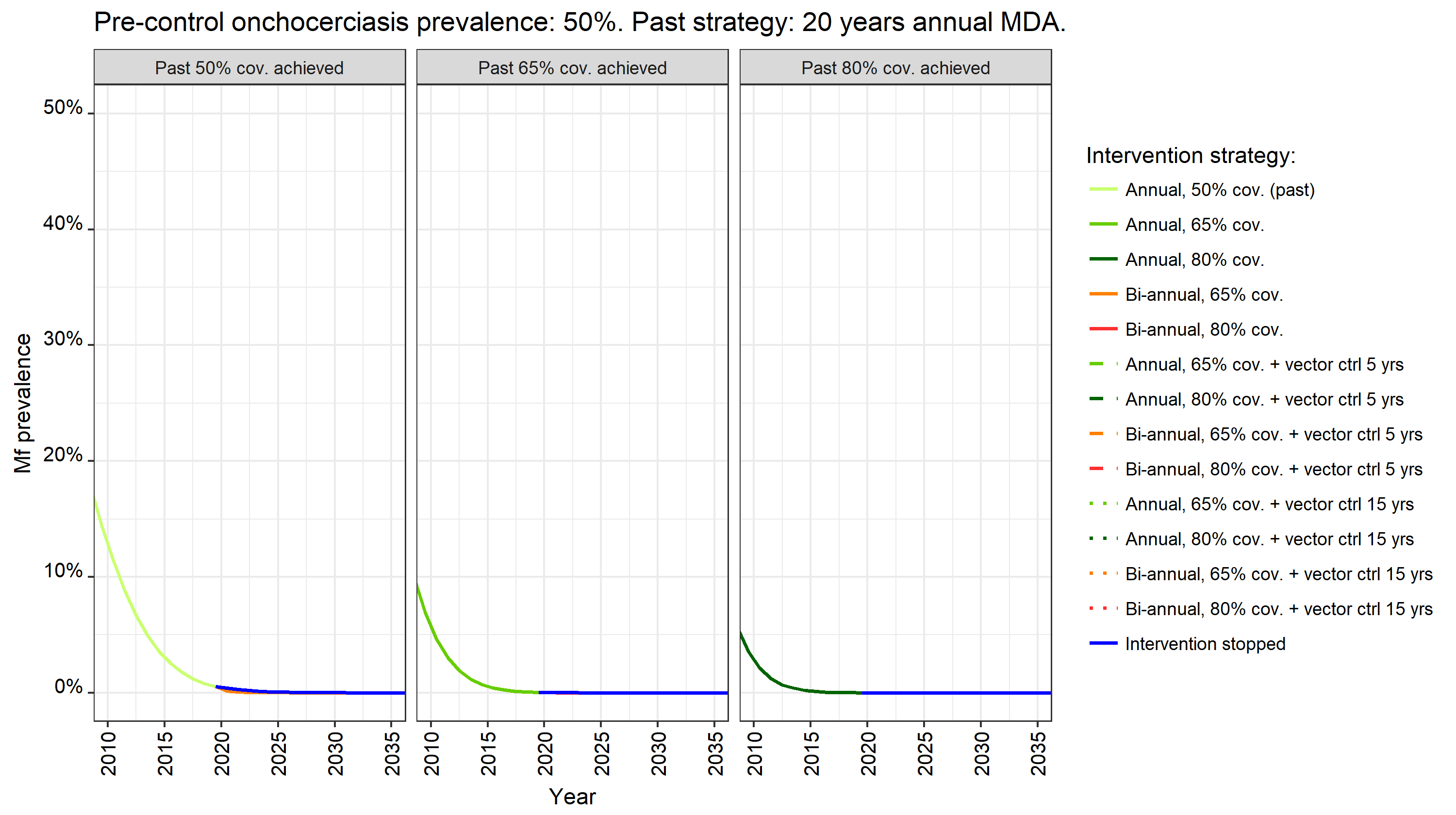

**Figure 2.5**

## Past 5 years bi-annual MDA.


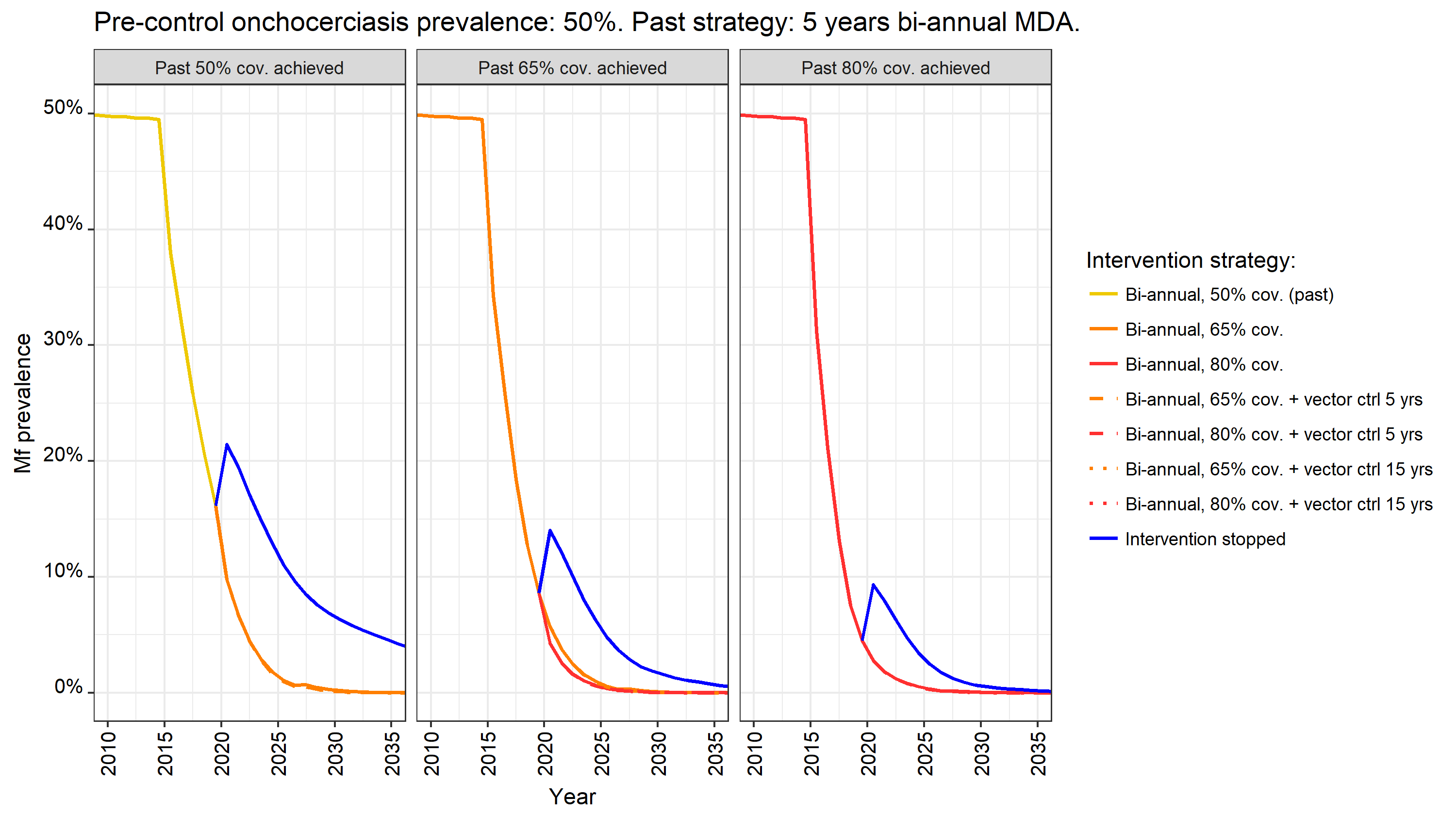

**Figure 2.6**

# Historic prevalence: 60%.

## Treatment naive.


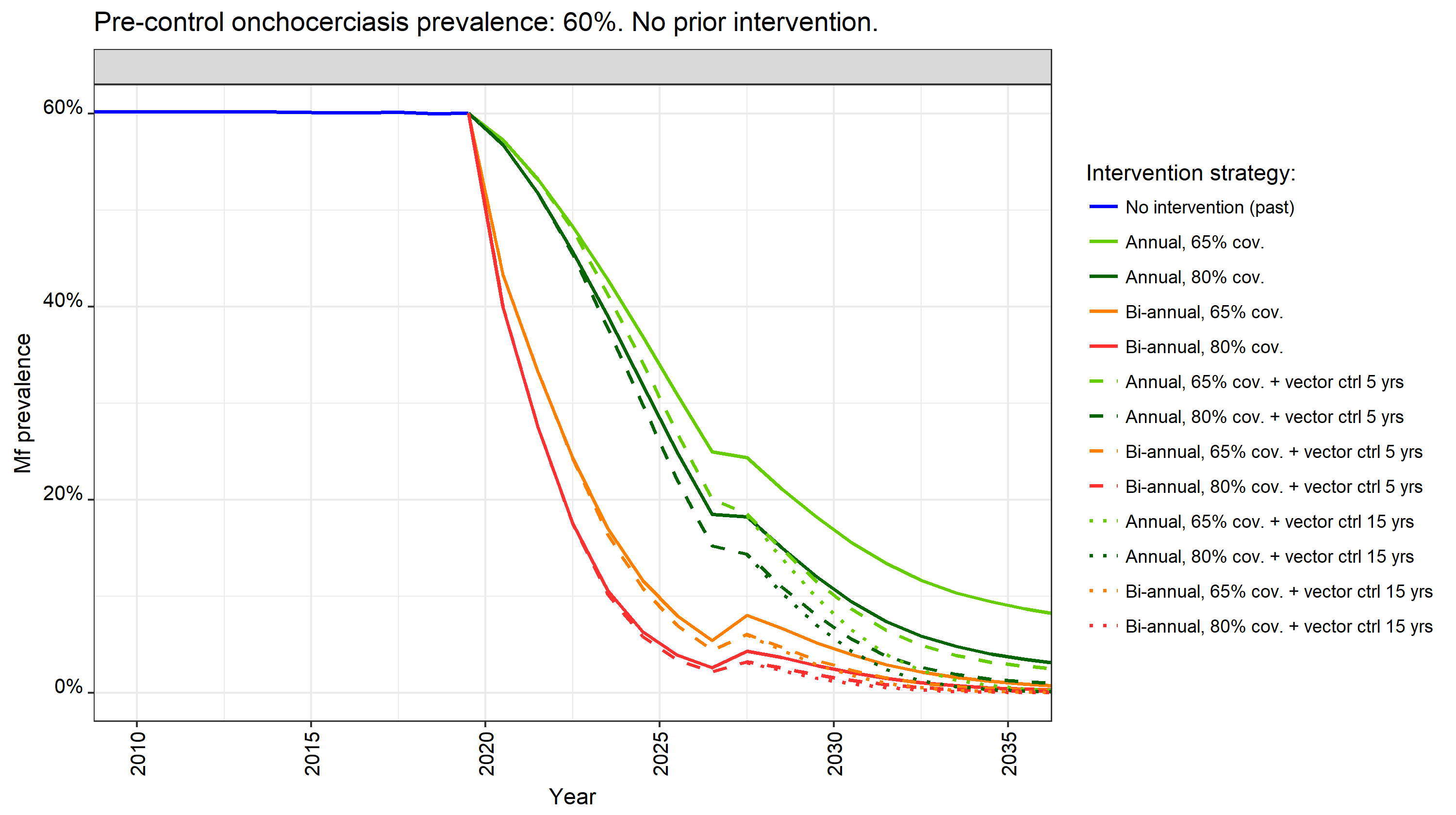

**Figure 3.1**

## Past 5 years annual MDA.


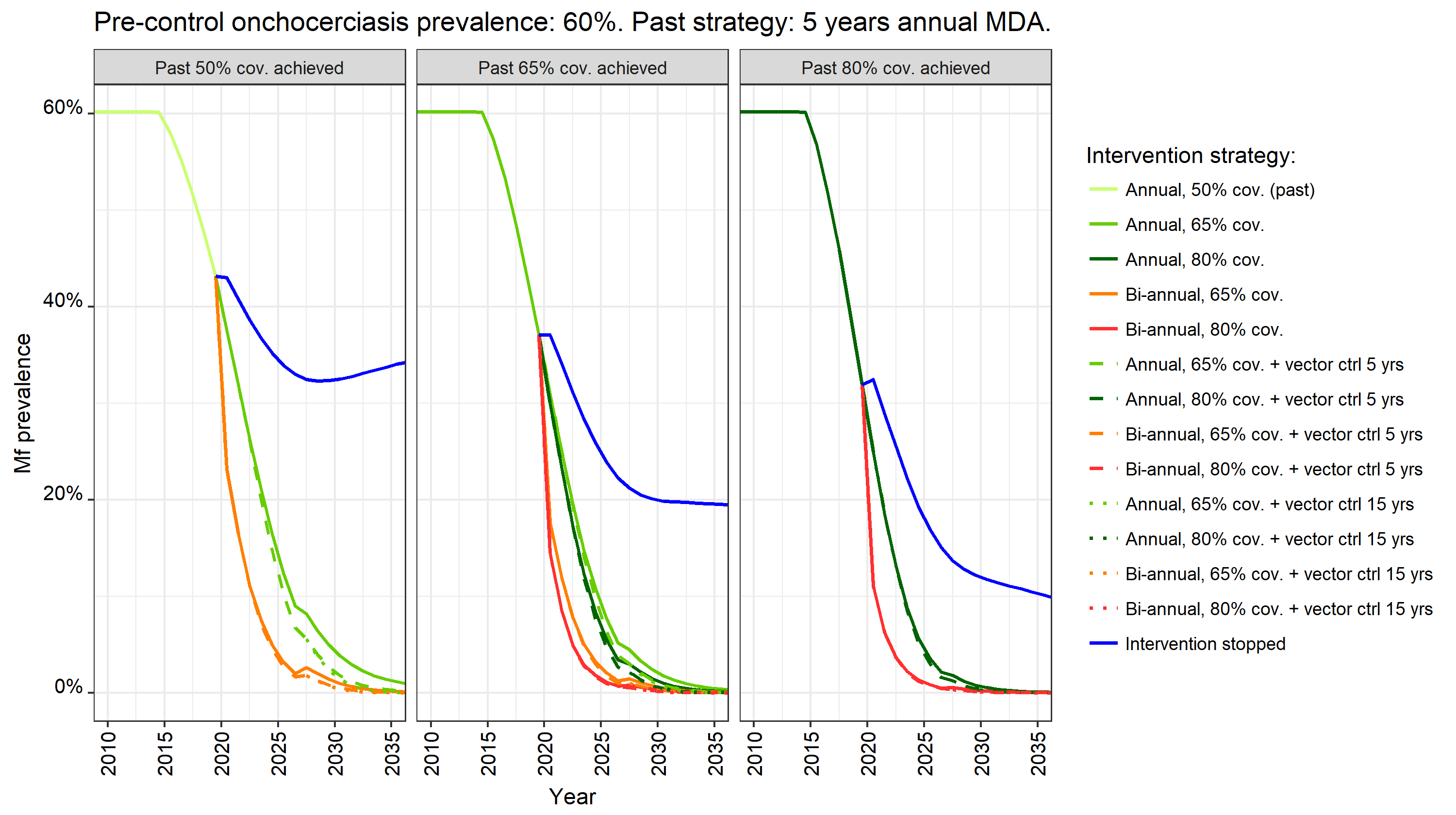

**Figure 3.2**

## Past 10 years annual MDA.


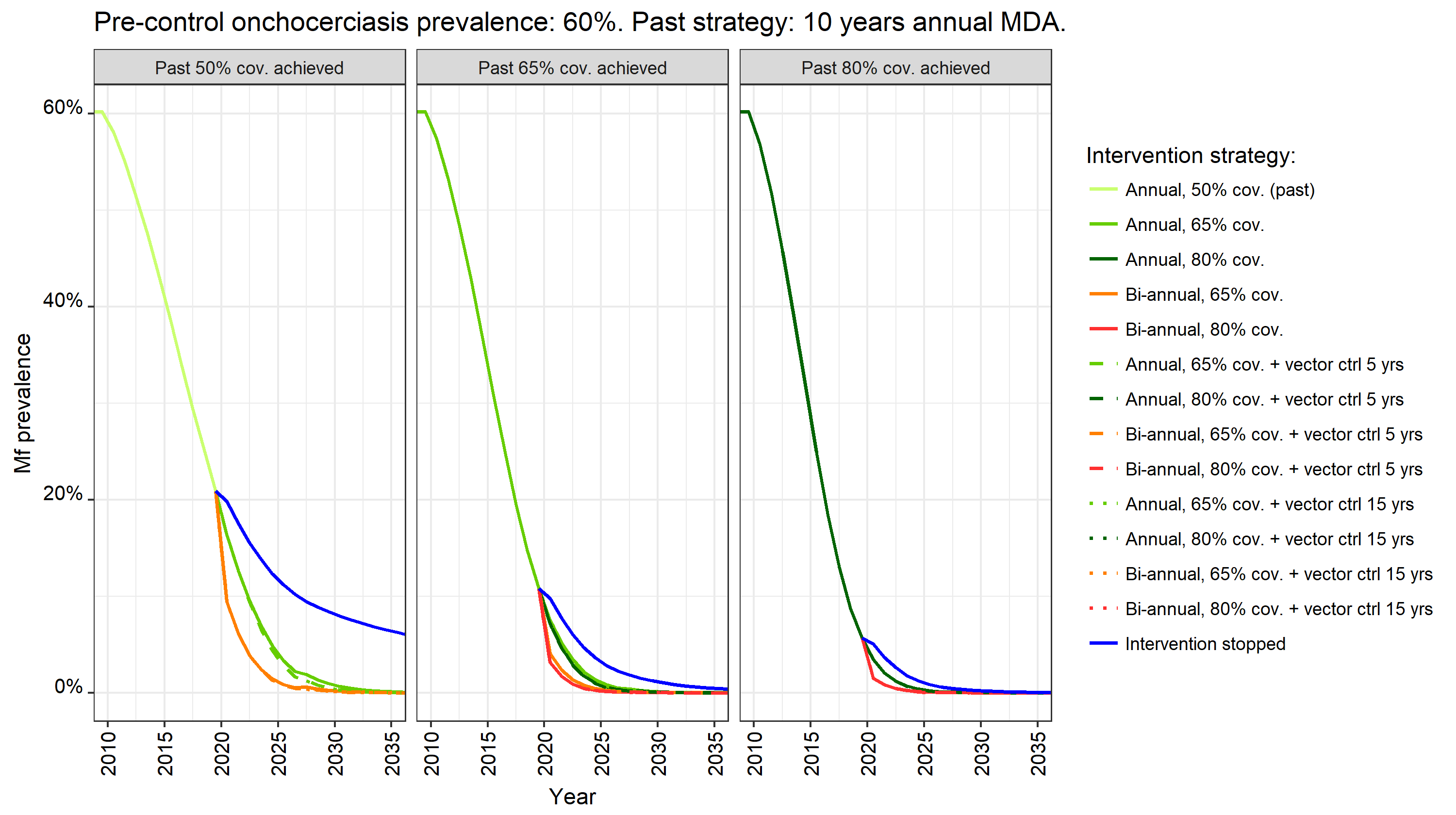

**Figure 3.3**

## Past 15 years annual MDA.


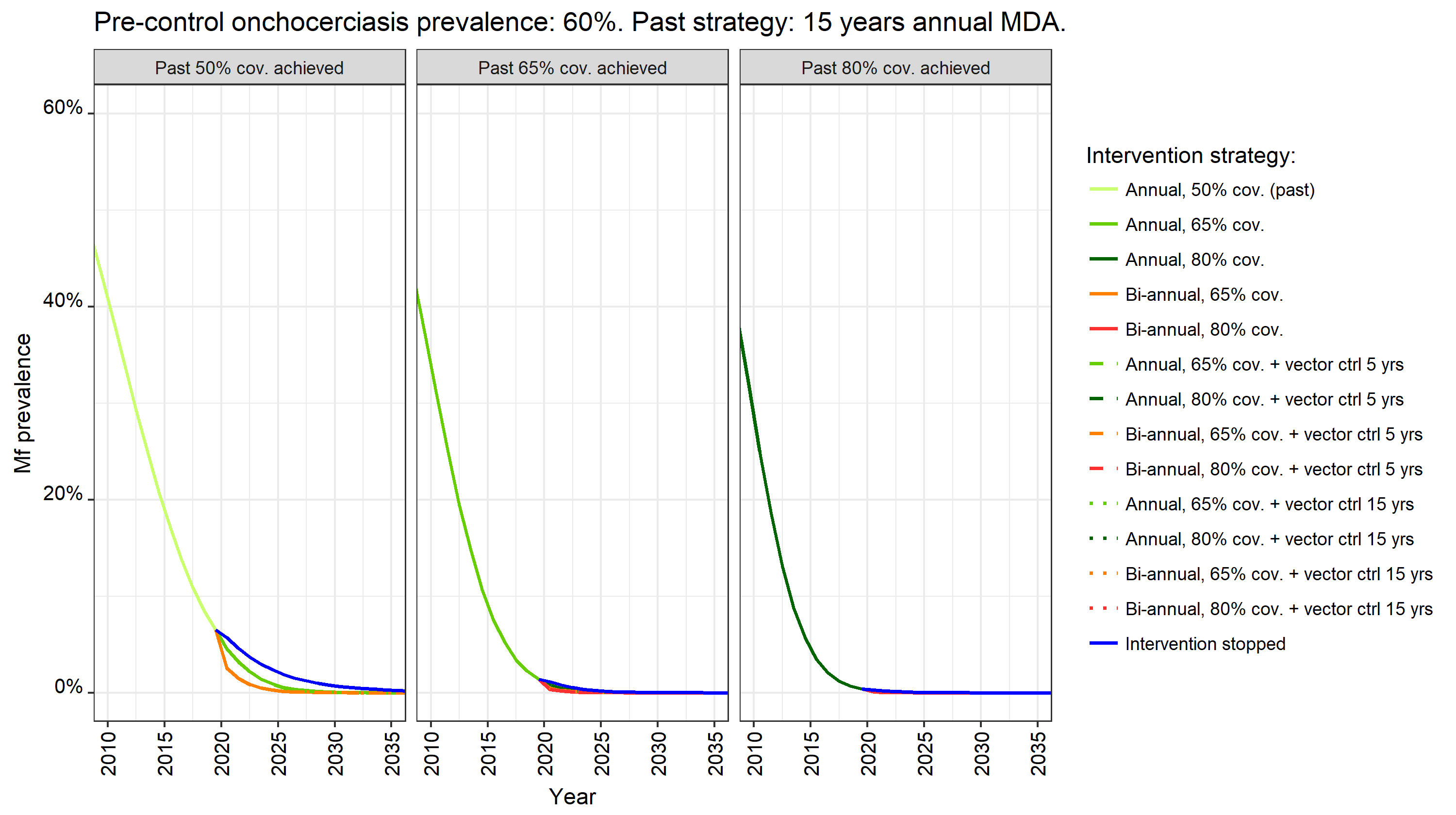

**Figure 3.4**

## Past 20 years annual MDA.


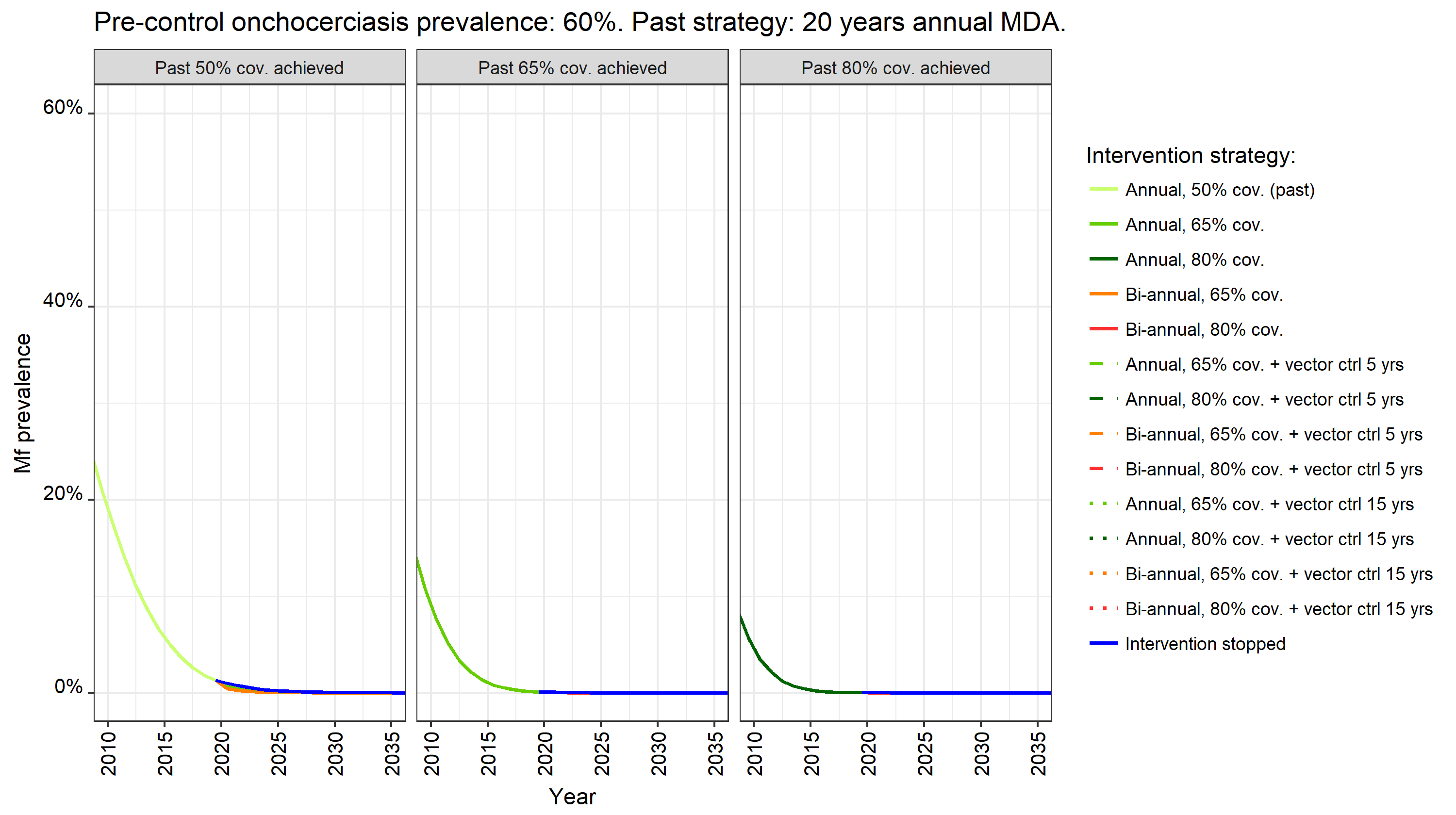

**Figure 3.5**

## Past 5 years bi-annual MDA.


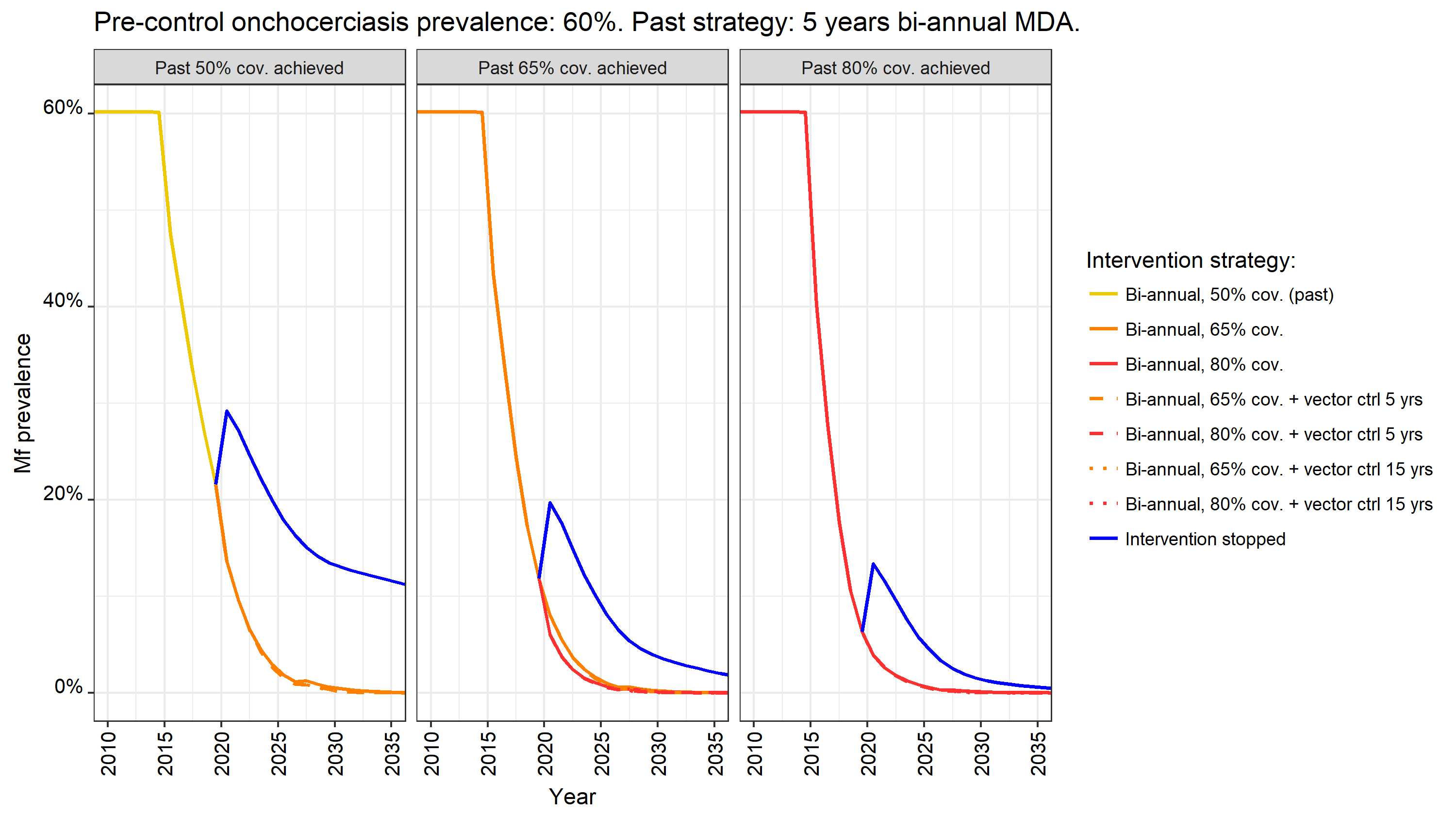

**Figure 3.6**

# Historic prevalence: 70%.

## Treatment naive.


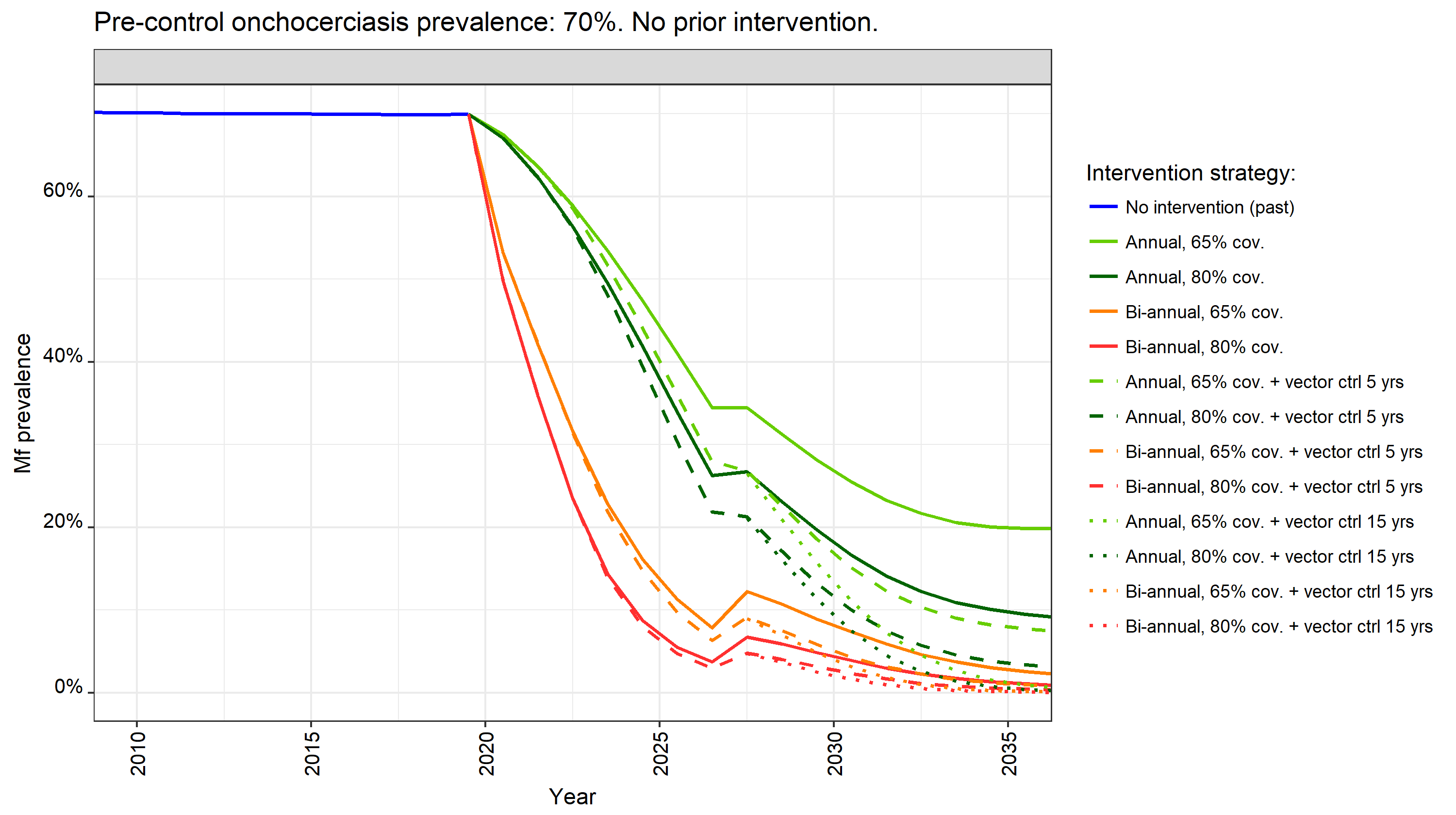

**Figure 4.1**

## Past 5 years annual MDA.


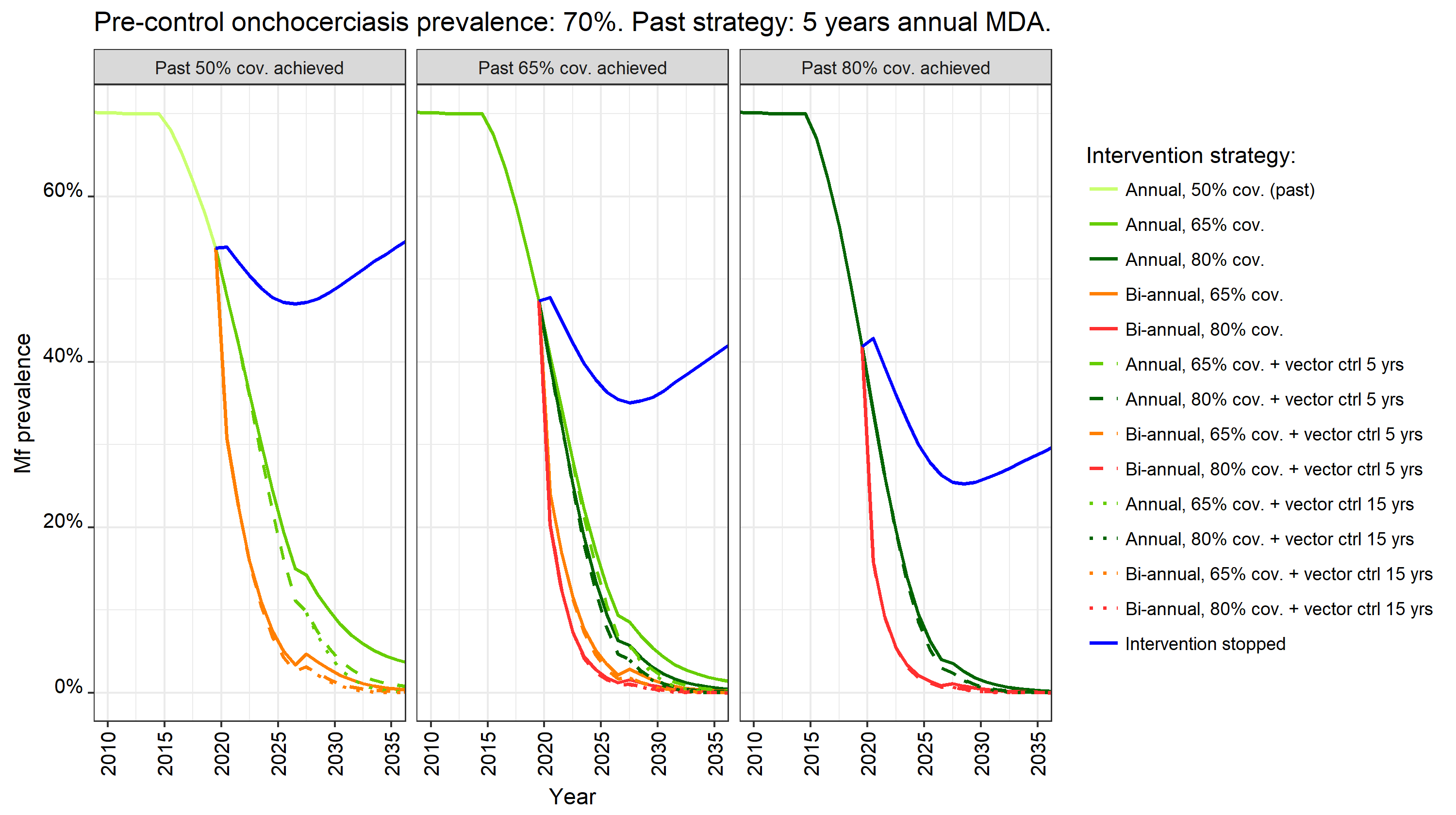

**Figure 4.2**

## Past 10 years annual MDA.


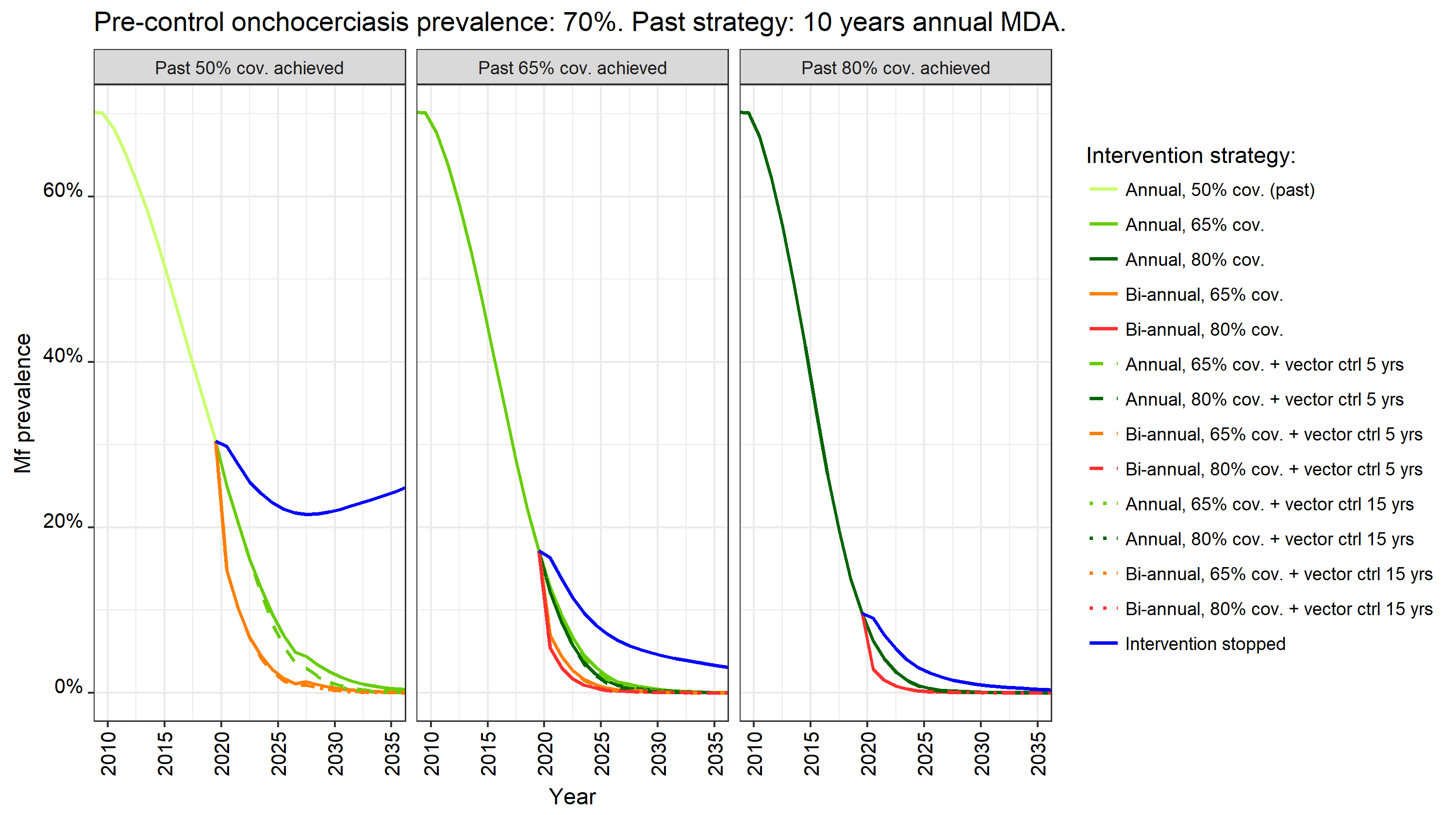

**Figure 4.3**

## Past 15 years annual MDA.


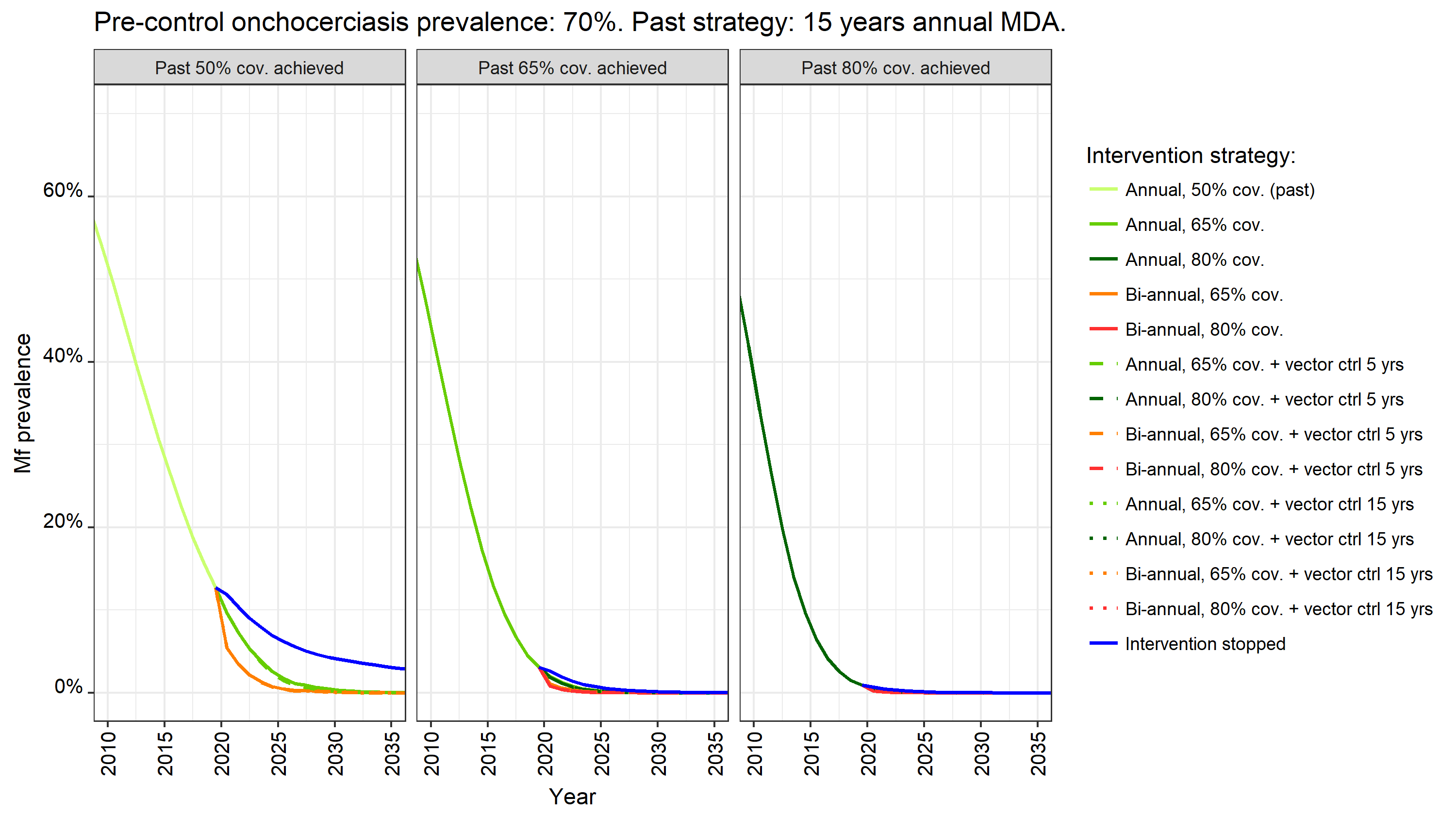

**Figure 4.4**

## Past 20 years annual MDA.


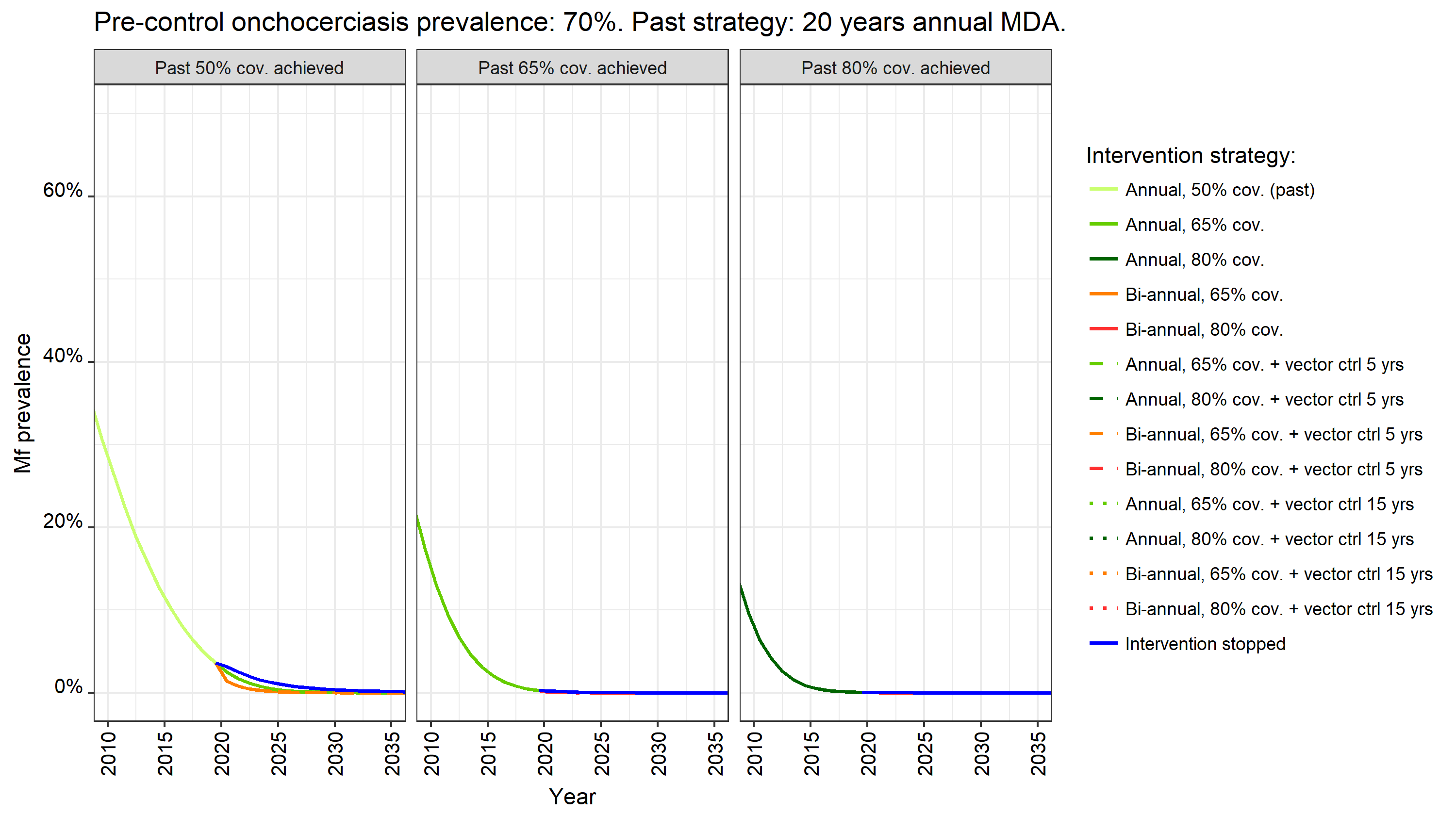

**Figure 4.5**

## Past 5 years bi-annual MDA.


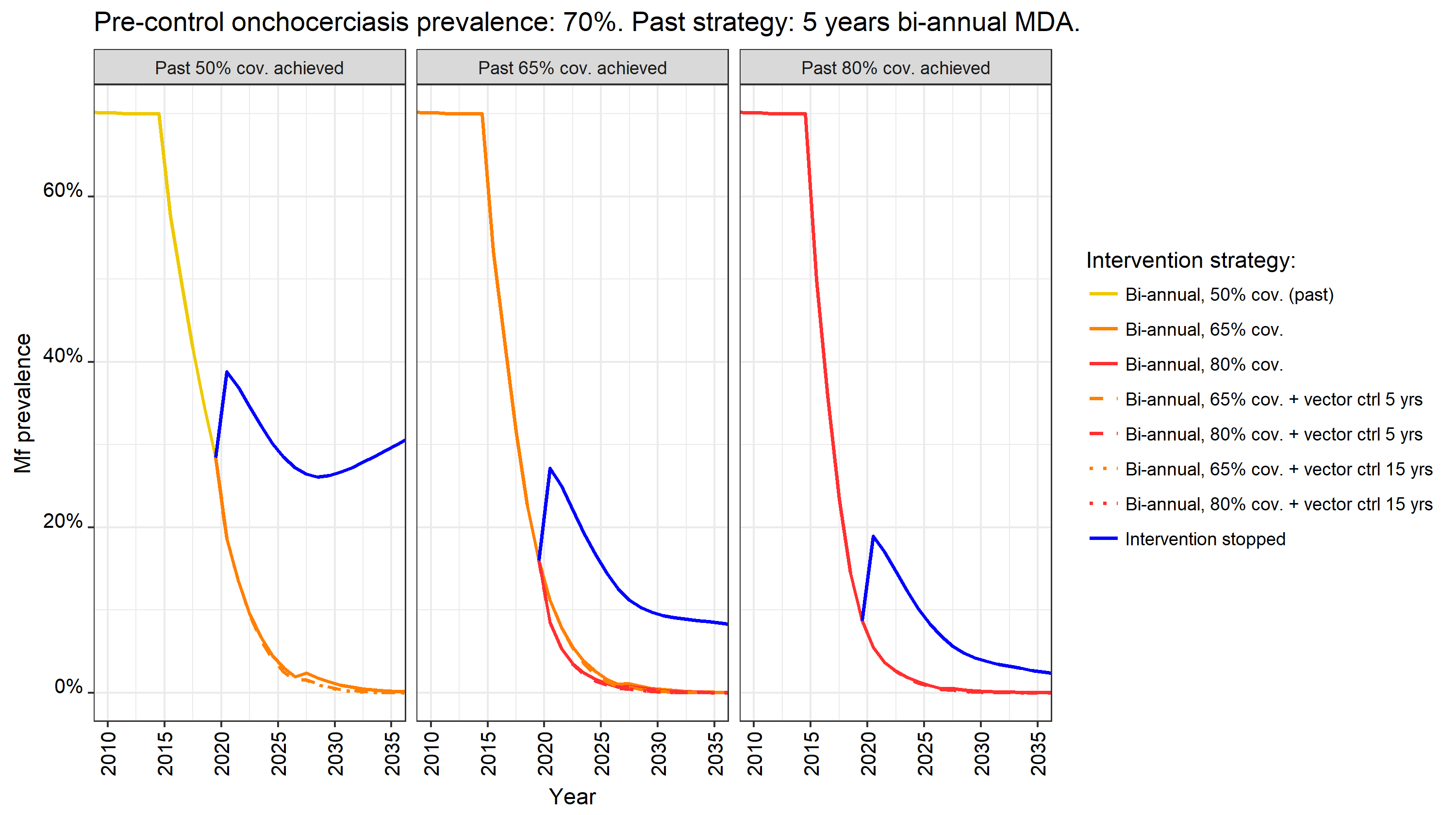

**Figure 4.6**

# Historic prevalence: 80%.

## Treatment naive.


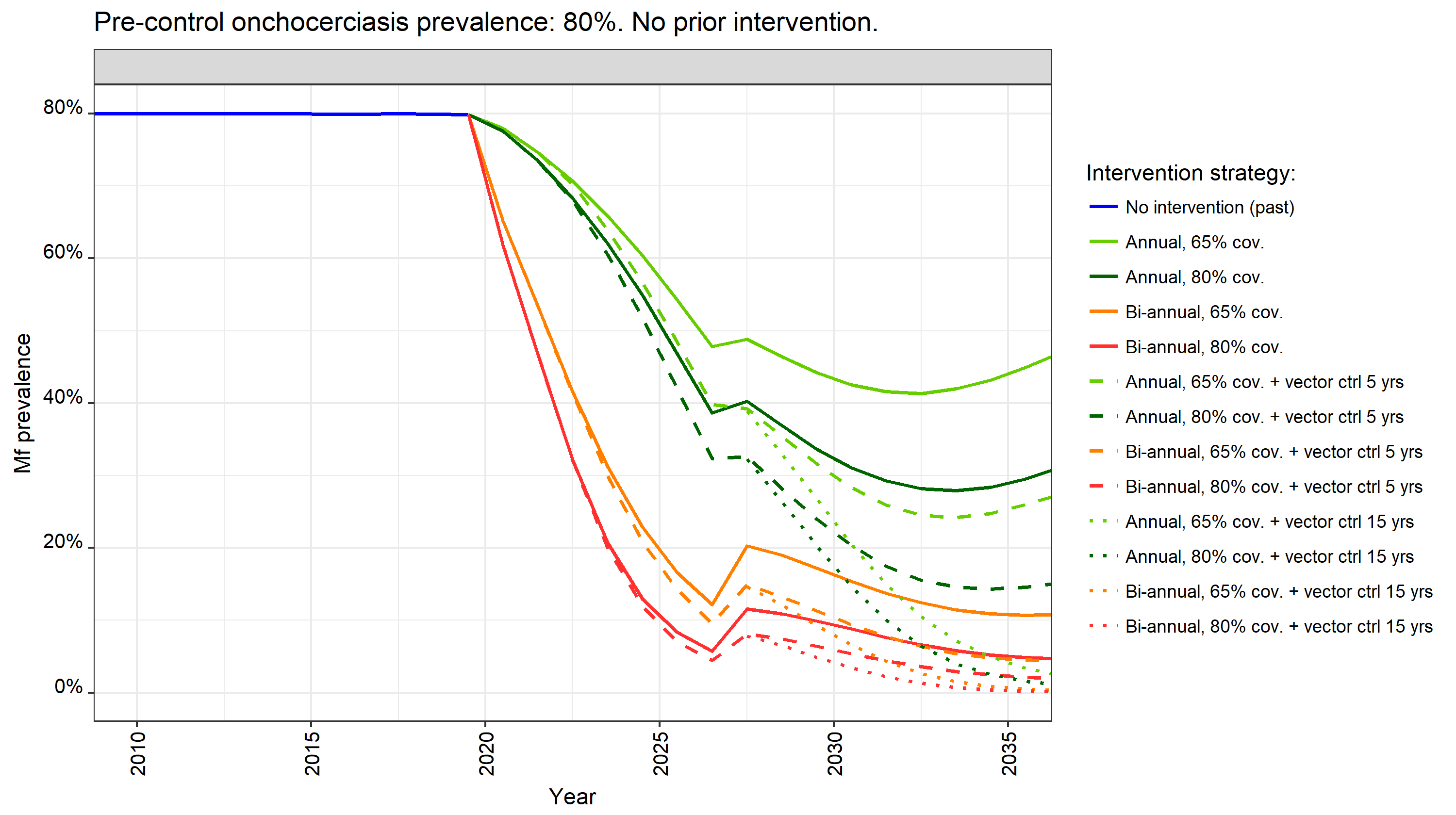

**Figure 5.1**

## Past 5 years annual MDA.


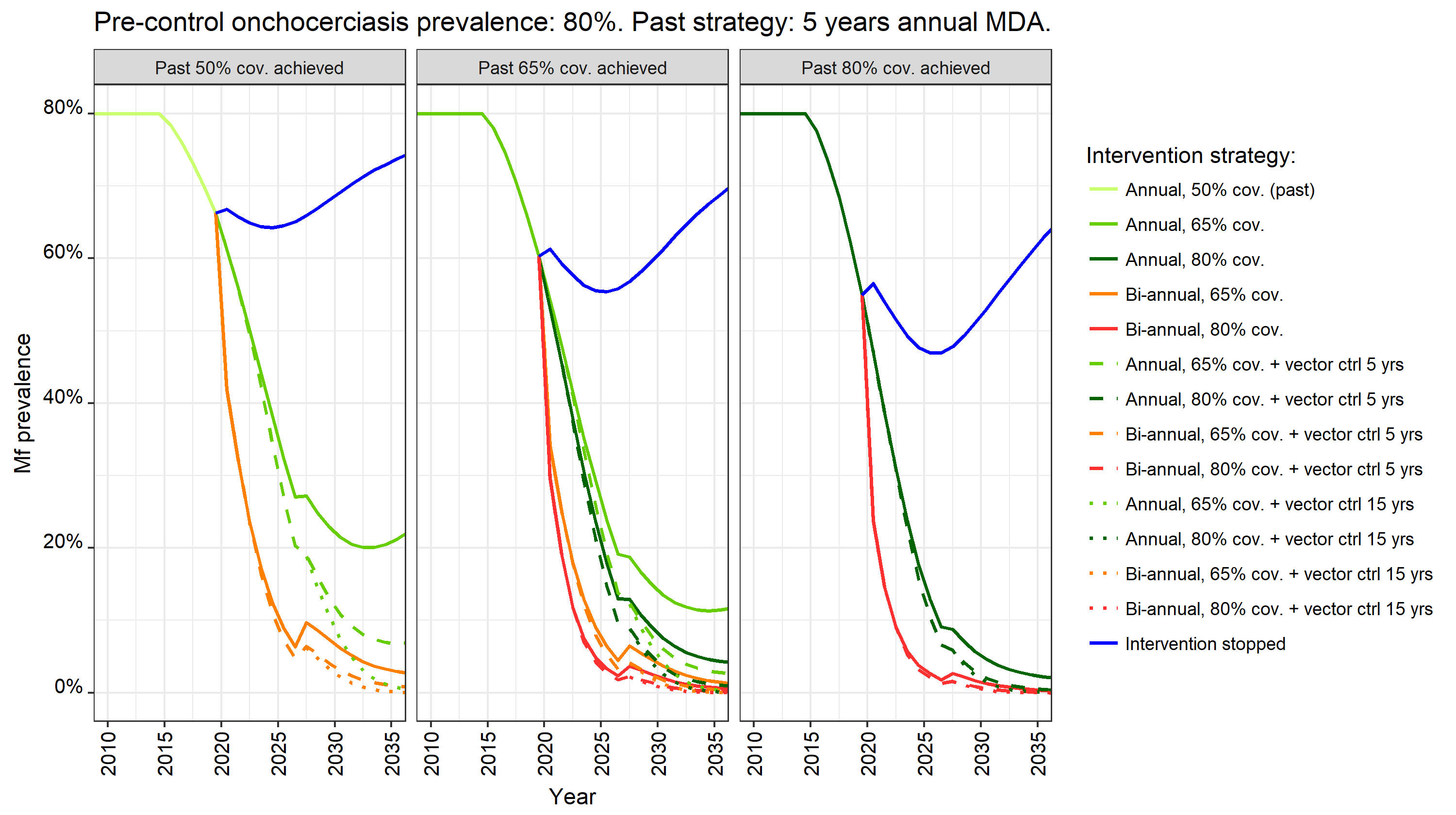

**Figure 5.2**

## Past 10 years annual MDA.


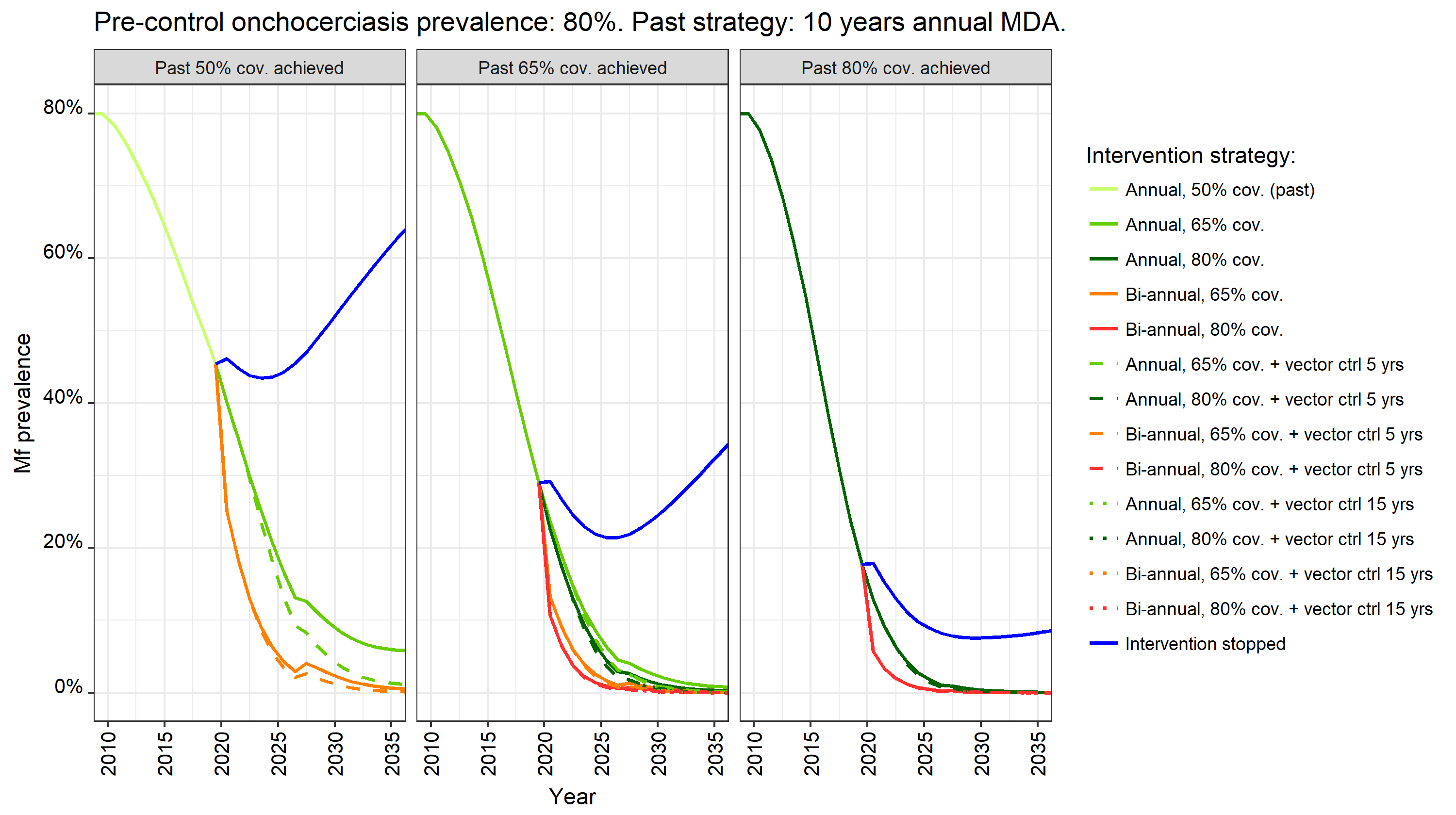

**Figure 5.3**

## Past 15 years annual MDA.


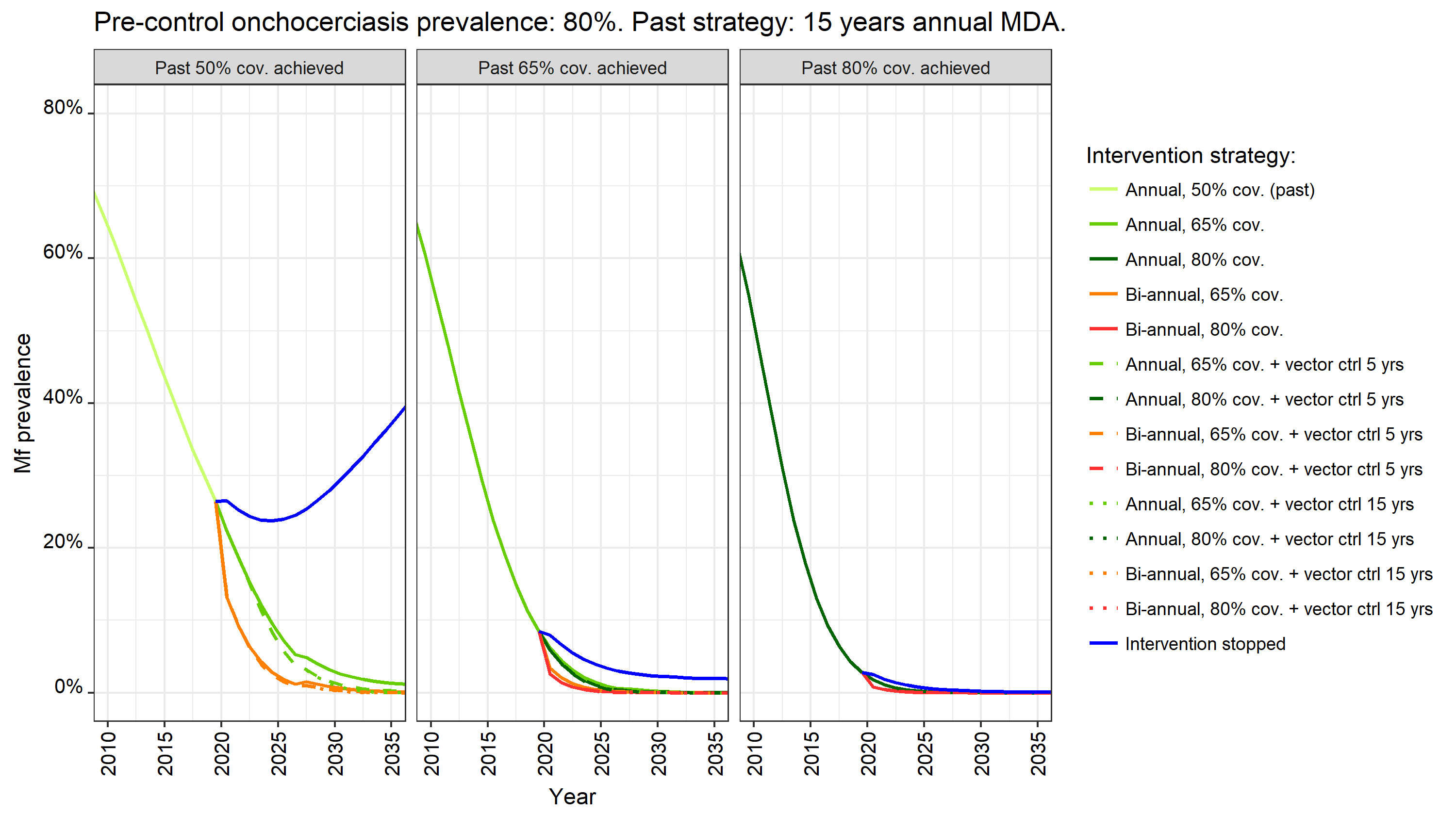

**Figure 5.4**

## Past 20 years annual MDA.


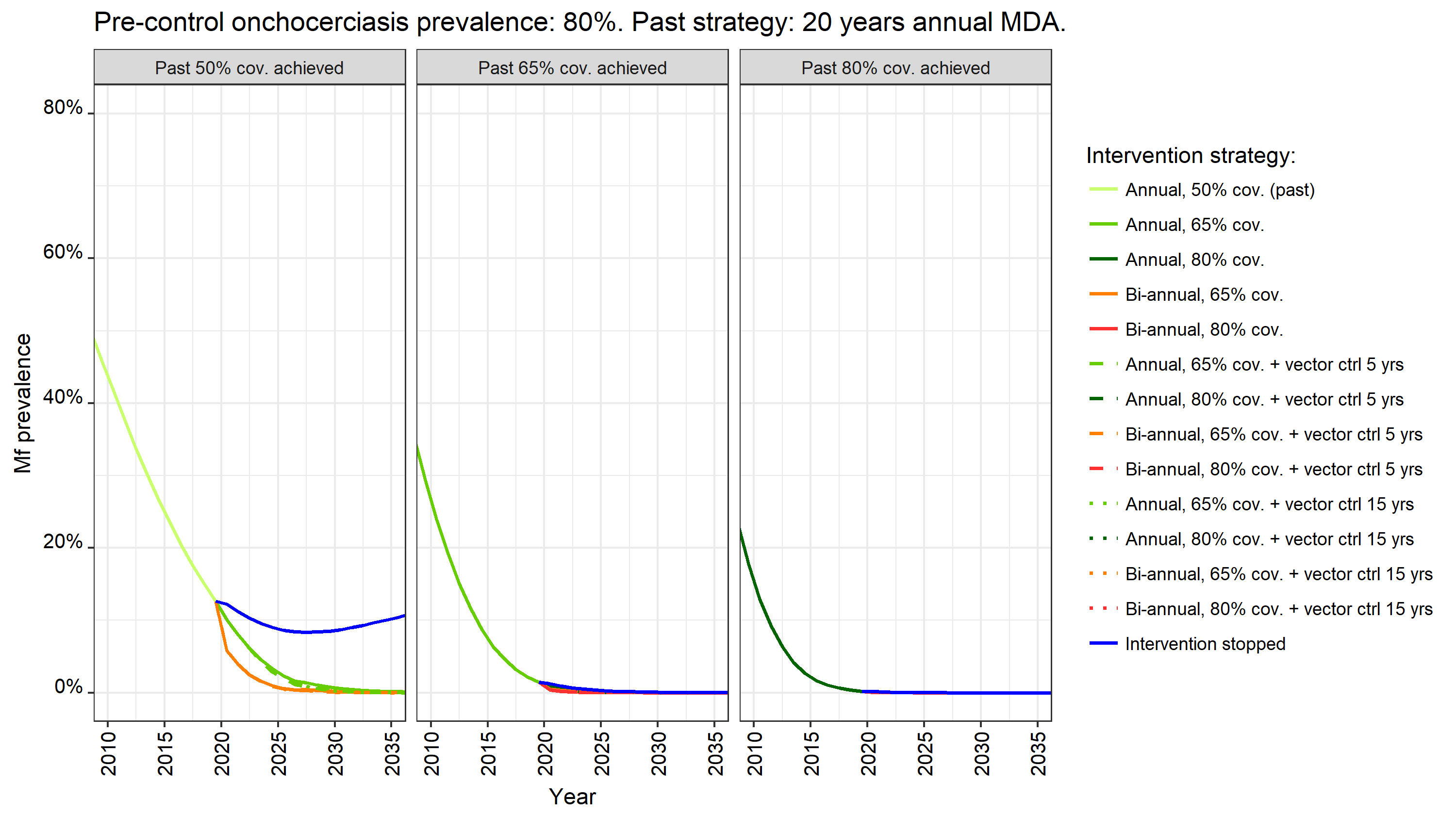

**Figure 5.5**

## Past 5 years bi-annual MDA.


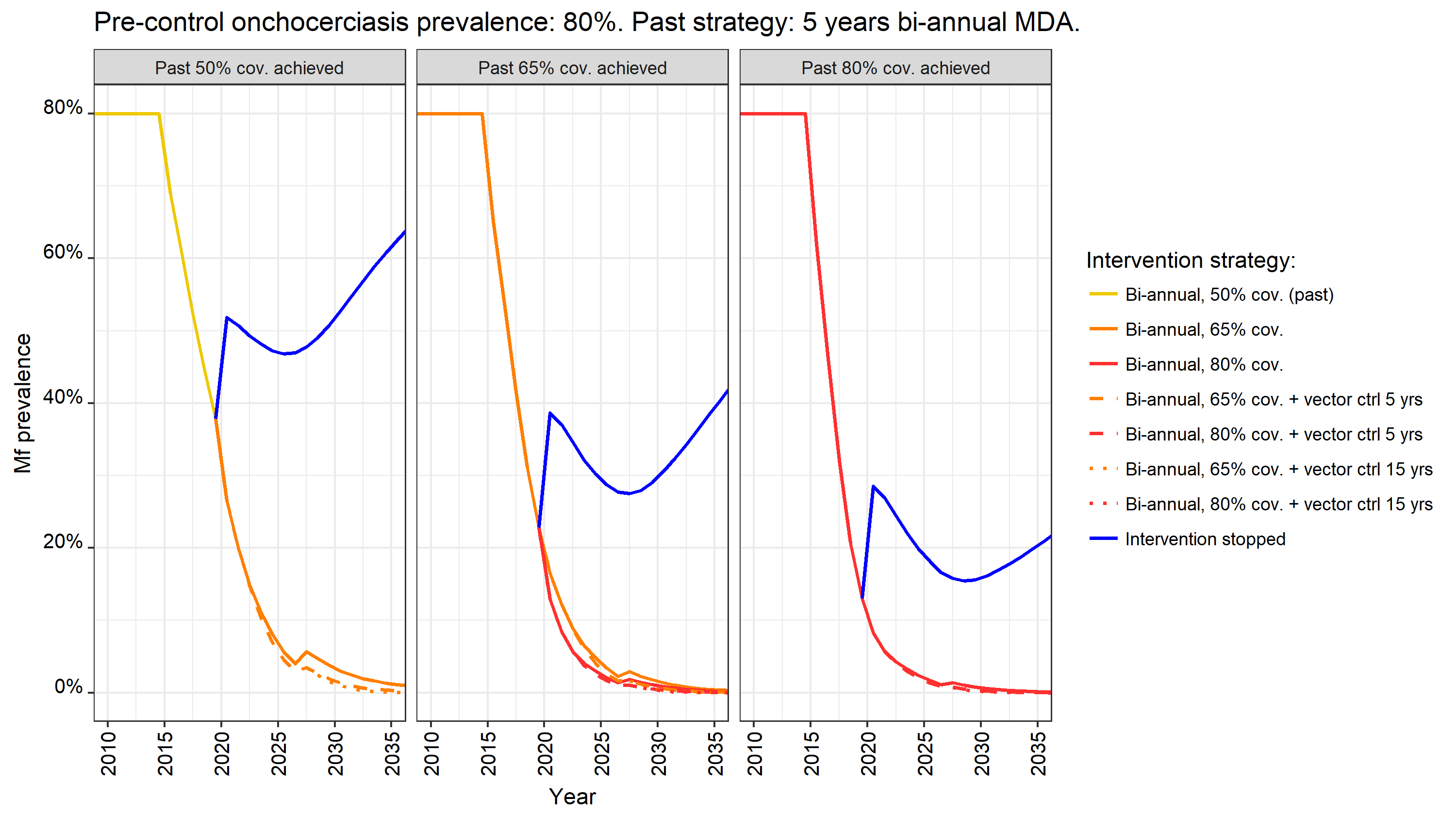

**Figure 5.6**
