# Supplementary material for: How Can Onchocerciasis Elimination in Africa Be Accelerated? Modeling the Impact of Increased Ivermectin Treatment Frequency and Complementary Vector Control
Source: Clin Infect Dis. 2018 Jun 1;66(Suppl 4):S267–74. doi: 10.1093/cid/cix1137 (PMC5982715; doi:10.1093/cid/cix1137)
Supplement: Supplementary Part 3 [file cix1137_suppl_supplementary_part_3.docx]

**How can onchocerciasis elimination in Africa be accelerated? Modelling the impact of increased ivermectin treatment frequency and complementary vector control**

Suzanne Verver, Martin Walker, Young Eun Kim, Grace Fobi, Afework H. Tekle, Honorat G.M. Zouré, Samuel Wanji, Daniel A. Boakye, Annette C. Kuesel, Sake J. de Vlas, Michel Boussinesq, Maria-Gloria Basáñez, Wilma A. Stolk

**Online Supplement 3: EPIONCHO figures**

Table of Contents

[1 Introduction to the supplement 4](#_Toc500496047)

[2 Pre-intervention microfilarial prevalence of 40% 4](#_Toc500496048)

[**Figure 2.1** Simulated dynamics between 2015 and 2035 in the microfilarial prevalence among individuals aged ≥ 5 years in settings with a 40% pre-control prevalence and no history of prior control. 4](#_Toc500496049)

[**Figure 2.2** Simulated dynamics between 2015 and 2035 in the microfilarial prevalence among individuals aged ≥ 5 years in settings with a 40% pre-control prevalence and a 5-year history of annual mass drug administration (MDA). 5](#_Toc500496050)

[**Figure 2.3** Simulated dynamics between 2015 and 2035 in the microfilarial prevalence among individuals aged ≥ 5 years in settings with a 40% pre-control prevalence and a 10-year history of annual mass drug administration (MDA). 5](#_Toc500496051)

[**Figure 2.4** Simulated dynamics between 2015 and 2035 in the microfilarial prevalence among individuals aged ≥ 5 years in settings with a 40% pre-control prevalence and a 15-year history of annual mass drug administration (MDA). 6](#_Toc500496052)

[**Figure 2.5** Simulated dynamics between 2015 and 2035 in the microfilarial prevalence among individuals aged ≥ 5 years in settings with a 40% pre-control prevalence and a 20-year history of annual mass drug administration (MDA). 6](#_Toc500496053)

[**Figure 2.6** Simulated dynamics between 2015 and 2035 in the microfilarial prevalence among individuals aged ≥ 5 years in settings with a 40% pre-control prevalence and a 5-year history of biannual mass drug administration (MDA). 7](#_Toc500496054)

[3 Pre-intervention microfilarial prevalence of 50% 7](#_Toc500496055)

[**Figure 3.1** Simulated dynamics between 2015 and 2035 in the microfilarial prevalence among individuals aged ≥ 5 years in settings with a 50% pre-control prevalence and no history of prior control. 7](#_Toc500496056)

[**Figure 3.2** Simulated dynamics between 2015 and 2035 in the microfilarial prevalence among individuals aged ≥ 5 years in settings with a 50% pre-control prevalence and a 5-year history of annual mass drug administration (MDA). 8](#_Toc500496057)

[**Figure 3.3** Simulated dynamics between 2015 and 2035 in the microfilarial prevalence among individuals aged ≥ 5 years in settings with a 50% pre-control prevalence and a 10-year history of annual mass drug administration (MDA). 8](#_Toc500496058)

[**Figure 3.4** Simulated dynamics between 2015 and 2035 in the microfilarial prevalence among individuals aged ≥ 5 years in settings with a 50% pre-control prevalence and a 15-year history of annual mass drug administration (MDA). 9](#_Toc500496059)

[**Figure 3.5** Simulated dynamics between 2015 and 2035 in the microfilarial prevalence among individuals aged ≥ 5 years in settings with a 50% pre-control prevalence and a 20-year history of annual mass drug administration (MDA). 9](#_Toc500496060)

[**Figure 3.6** Simulated dynamics between 2015 and 2035 in the microfilarial prevalence among individuals aged ≥ 5 years in settings with a 50% pre-control prevalence and a 5-year history of biannual mass drug administration (MDA). 10](#_Toc500496061)

[4 Pre-intervention microfilarial prevalence of 60% 10](#_Toc500496062)

[**Figure 4.1** Simulated dynamics between 2015 and 2035 in the microfilarial prevalence among individuals aged ≥ 5 years in settings with a 60% pre-control prevalence and no history of prior control. 10](#_Toc500496063)

[**Figure 4.2** Simulated dynamics between 2015 and 2035 in the microfilarial prevalence among individuals aged ≥ 5 years in settings with a 60% pre-control prevalence and a 5-year history of annual mass drug administration (MDA). 11](#_Toc500496064)

[**Figure 4.3** Simulated dynamics between 2015 and 2035 in the microfilarial prevalence among individuals aged ≥ 5 years in settings with a 60% pre-control prevalence and a 10-year history of annual mass drug administration (MDA). 11](#_Toc500496065)

[**Figure 4.4** Simulated dynamics between 2015 and 2035 in the microfilarial prevalence among individuals aged ≥ 5 years in settings with a 60% pre-control prevalence and a 15-year history of annual mass drug administration (MDA). 12](#_Toc500496066)

[**Figure 4.5** Simulated dynamics between 2015 and 2035 in the microfilarial prevalence among individuals aged ≥ 5 years in settings with a 60% pre-control prevalence and a 20-year history of annual mass drug administration (MDA). 12](#_Toc500496067)

[**Figure 4.6** Simulated dynamics between 2015 and 2035 in the microfilarial prevalence among individuals aged ≥ 5 years in settings with a 60% pre-control prevalence and a 5-year history of biannual mass drug administration (MDA). 13](#_Toc500496068)

[5 Pre-intervention microfilarial prevalence of 70% 13](#_Toc500496069)

[**Figure 5.1** Simulated dynamics between 2015 and 2035 in the microfilarial prevalence among individuals aged ≥ 5 years in settings with a 70% pre-control prevalence and no history of prior control. 13](#_Toc500496070)

[**Figure 5.2** Simulated dynamics between 2015 and 2035 in the microfilarial prevalence among individuals aged ≥ 5 years in settings with a 70% pre-control prevalence and a 5-year history of annual mass drug administration (MDA). 14](#_Toc500496071)

[**Figure 5.3** Simulated dynamics between 2015 and 2035 in the microfilarial prevalence among individuals aged ≥ 5 years in settings with a 70% pre-control prevalence and a 10-year history of annual mass drug administration (MDA). 14](#_Toc500496072)

[**Figure 5.4** Simulated dynamics between 2015 and 2035 in the microfilarial prevalence among individuals aged ≥ 5 years in settings with a 70% pre-control prevalence and a 15-year history of annual mass drug administration (MDA). 15](#_Toc500496073)

[**Figure 5.5** Simulated dynamics between 2015 and 2035 in the microfilarial prevalence among individuals aged ≥ 5 years in settings with a 70% pre-control prevalence and a 20-year history of annual mass drug administration (MDA). 15](#_Toc500496074)

[**Figure 5.6** Simulated dynamics between 2015 and 2035 in the microfilarial prevalence among individuals aged ≥ 5 years in settings with a 70% pre-control prevalence and a 5-year history of biannual mass drug administration (MDA). 16](#_Toc500496075)

[6 Pre-intervention microfilarial prevalence of 80% 16](#_Toc500496076)

[**Figure 6.1** Simulated dynamics between 2015 and 2035 in the microfilarial prevalence among individuals aged ≥ 5 years in settings with a 80% pre-control prevalence and no history of prior control. 16](#_Toc500496077)

[**Figure 6.2** Simulated dynamics between 2015 and 2035 in the microfilarial prevalence among individuals aged ≥ 5 years in settings with a 80% pre-control prevalence and a 5-year history of annual mass drug administration (MDA). 17](#_Toc500496078)

[**Figure 6.3** Simulated dynamics between 2015 and 2035 in the microfilarial prevalence among individuals aged ≥ 5 years in settings with a 80% pre-control prevalence and a 10-year history of annual mass drug administration (MDA). 17](#_Toc500496079)

[**Figure 6.4** Simulated dynamics between 2015 and 2035 in the microfilarial prevalence among individuals aged ≥ 5 years in settings with a 80% pre-control prevalence and a 15-year history of annual mass drug administration (MDA). 18](#_Toc500496080)

[**Figure 6.5** Simulated dynamics between 2015 and 2035 in the microfilarial prevalence among individuals aged ≥ 5 years in settings with a 80% pre-control prevalence and a 20-year history of annual mass drug administration (MDA). 18](#_Toc500496081)

[**Figure 6.6** Simulated dynamics between 2015 and 2035 in the microfilarial prevalence among individuals aged ≥ 5 years in settings with a 80% pre-control prevalence and a 5-year history of biannual mass drug administration (MDA). 19](#_Toc500496082)

# Introduction to the supplement

Each figure depicts the microfilarial prevalence dynamics until 2035 in individuals aged ≥ 5 years during simulated interventions against onchocerciasis in different endemicity settings before and after a switch to a different intervention strategy implemented from 2019 to 2025 (excepting vector control implemented for 15 years which continued until 2035). Each figure depicts the simulated dynamics in settings with a pre-control microfilarial prevalence of 40, 50, 60, 70 or 80% with different histories of past control and future scenarios of no mass drug administration (MDA), annual MDA for 5,10,15 or 20 years, biannual MDA for 5 years with or without 5 or 15 years of complementary vector control. The dynamics elicited by quarterly MDA are not shown to prevent crowding of the figures.

# Pre-intervention microfilarial prevalence of 40%

**Figure 2.1** Simulated dynamics between 2015 and 2035 in the microfilarial prevalence among individuals aged ≥ 5 years in settings with a 40% pre-control prevalence and no history of prior control. Future intervention scenarios implemented from 2019 (mass drug administration) or 2021 (vector control) are depicted by different colored lines as indicated in the legend.

**Figure 2.2** Simulated dynamics between 2015 and 2035 in the microfilarial prevalence among individuals aged ≥ 5 years in settings with a 40% pre-control prevalence and a 5-year history of annual mass drug administration (MDA). Future intervention scenarios implemented from 2019 (MDA) or 2021 (vector control) are depicted by different colored lines as indicated in the legend.

**Figure 2.3** Simulated dynamics between 2015 and 2035 in the microfilarial prevalence among individuals aged ≥ 5 years in settings with a 40% pre-control prevalence and a 10-year history of annual mass drug administration (MDA). Future intervention scenarios implemented from 2019 (MDA) or 2021 (vector control) are depicted by different colored lines as indicated in the legend.

**Figure 2.4** Simulated dynamics between 2015 and 2035 in the microfilarial prevalence among individuals aged ≥ 5 years in settings with a 40% pre-control prevalence and a 15-year history of annual mass drug administration (MDA). Future intervention scenarios implemented from 2019 (MDA) or 2021 (vector control) are depicted by different colored lines as indicated in the legend.

**Figure 2.5** Simulated dynamics between 2015 and 2035 in the microfilarial prevalence among individuals aged ≥ 5 years in settings with a 40% pre-control prevalence and a 20-year history of annual mass drug administration (MDA). Future intervention scenarios implemented from 2019 (MDA) or 2021 (vector control) are depicted by different colored lines as indicated in the legend.

**Figure 2.6** Simulated dynamics between 2015 and 2035 in the microfilarial prevalence among individuals aged ≥ 5 years in settings with a 40% pre-control prevalence and a 5-year history of biannual mass drug administration (MDA). Future intervention scenarios implemented from 2019 (MDA) or 2021 (vector control) are depicted by different colored lines as indicated in the legend.

# Pre-intervention microfilarial prevalence of 50%

**Figure 3.1** Simulated dynamics between 2015 and 2035 in the microfilarial prevalence among individuals aged ≥ 5 years in settings with a 50% pre-control prevalence and no history of prior control. Future intervention scenarios implemented from 2019 (mass drug administration) or 2021 (vector control) are depicted by different colored lines as indicated in the legend.

**Figure 3.2** Simulated dynamics between 2015 and 2035 in the microfilarial prevalence among individuals aged ≥ 5 years in settings with a 50% pre-control prevalence and a 5-year history of annual mass drug administration (MDA). Future intervention scenarios implemented from 2019 (MDA) or 2021 (vector control) are depicted by different colored lines as indicated in the legend.

**Figure 3.3** Simulated dynamics between 2015 and 2035 in the microfilarial prevalence among individuals aged ≥ 5 years in settings with a 50% pre-control prevalence and a 10-year history of annual mass drug administration (MDA). Future intervention scenarios implemented from 2019 (MDA) or 2021 (vector control) are depicted by different colored lines as indicated in the legend.

**Figure 3.4** Simulated dynamics between 2015 and 2035 in the microfilarial prevalence among individuals aged ≥ 5 years in settings with a 50% pre-control prevalence and a 15-year history of annual mass drug administration (MDA). Future intervention scenarios implemented from 2019 (MDA) or 2021 (vector control) are depicted by different colored lines as indicated in the legend.

**Figure 3.5** Simulated dynamics between 2015 and 2035 in the microfilarial prevalence among individuals aged ≥ 5 years in settings with a 50% pre-control prevalence and a 20-year history of annual mass drug administration (MDA). Future intervention scenarios implemented from 2019 (MDA) or 2021 (vector control) are depicted by different colored lines as indicated in the legend.

**Figure 3.6** Simulated dynamics between 2015 and 2035 in the microfilarial prevalence among individuals aged ≥ 5 years in settings with a 50% pre-control prevalence and a 5-year history of biannual mass drug administration (MDA). Future intervention scenarios implemented from 2019 (MDA) or 2021 (vector control) are depicted by different colored lines as indicated in the legend.

# Pre-intervention microfilarial prevalence of 60%

**Figure 4.1** Simulated dynamics between 2015 and 2035 in the microfilarial prevalence among individuals aged ≥ 5 years in settings with a 60% pre-control prevalence and no history of prior control. Future intervention scenarios implemented from 2019 (mass drug administration) or 2021 (vector control) are depicted by different colored lines as indicated in the legend.

**Figure 4.2** Simulated dynamics between 2015 and 2035 in the microfilarial prevalence among individuals aged ≥ 5 years in settings with a 60% pre-control prevalence and a 5-year history of annual mass drug administration (MDA). Future intervention scenarios implemented from 2019 (MDA) or 2021 (vector control) are depicted by different colored lines as indicated in the legend.

**Figure 4.3** Simulated dynamics between 2015 and 2035 in the microfilarial prevalence among individuals aged ≥ 5 years in settings with a 60% pre-control prevalence and a 10-year history of annual mass drug administration (MDA). Future intervention scenarios implemented from 2019 (MDA) or 2021 (vector control) are depicted by different colored lines as indicated in the legend.

**Figure 4.4** Simulated dynamics between 2015 and 2035 in the microfilarial prevalence among individuals aged ≥ 5 years in settings with a 60% pre-control prevalence and a 15-year history of annual mass drug administration (MDA). Future intervention scenarios implemented from 2019 (MDA) or 2021 (vector control) are depicted by different colored lines as indicated in the legend.

**Figure 4.5** Simulated dynamics between 2015 and 2035 in the microfilarial prevalence among individuals aged ≥ 5 years in settings with a 60% pre-control prevalence and a 20-year history of annual mass drug administration (MDA). Future intervention scenarios implemented from 2019 (MDA) or 2021 (vector control) are depicted by different colored lines as indicated in the legend.

**Figure 4.6** Simulated dynamics between 2015 and 2035 in the microfilarial prevalence among individuals aged ≥ 5 years in settings with a 60% pre-control prevalence and a 5-year history of biannual mass drug administration (MDA). Future intervention scenarios implemented from 2019 (MDA) or 2021 (vector control) are depicted by different colored lines as indicated in the legend.

# Pre-intervention microfilarial prevalence of 70%

**Figure 5.1** Simulated dynamics between 2015 and 2035 in the microfilarial prevalence among individuals aged ≥ 5 years in settings with a 70% pre-control prevalence and no history of prior control. Future intervention scenarios implemented from 2019 (mass drug administration) or 2021 (vector control) are depicted by different colored lines as indicated in the legend.

**Figure 5.2** Simulated dynamics between 2015 and 2035 in the microfilarial prevalence among individuals aged ≥ 5 years in settings with a 70% pre-control prevalence and a 5-year history of annual mass drug administration (MDA). Future intervention scenarios implemented from 2019 (MDA) or 2021 (vector control) are depicted by different colored lines as indicated in the legend.

**Figure 5.3** Simulated dynamics between 2015 and 2035 in the microfilarial prevalence among individuals aged ≥ 5 years in settings with a 70% pre-control prevalence and a 10-year history of annual mass drug administration (MDA). Future intervention scenarios implemented from 2019 (MDA) or 2021 (vector control) are depicted by different colored lines as indicated in the legend.

**Figure 5.4** Simulated dynamics between 2015 and 2035 in the microfilarial prevalence among individuals aged ≥ 5 years in settings with a 70% pre-control prevalence and a 15-year history of annual mass drug administration (MDA). Future intervention scenarios implemented from 2019 (MDA) or 2021 (vector control) are depicted by different colored lines as indicated in the legend.

**Figure 5.5** Simulated dynamics between 2015 and 2035 in the microfilarial prevalence among individuals aged ≥ 5 years in settings with a 70% pre-control prevalence and a 20-year history of annual mass drug administration (MDA). Future intervention scenarios implemented from 2019 (MDA) or 2021 (vector control) are depicted by different colored lines as indicated in the legend

**Figure 5.6** Simulated dynamics between 2015 and 2035 in the microfilarial prevalence among individuals aged ≥ 5 years in settings with a 70% pre-control prevalence and a 5-year history of biannual mass drug administration (MDA). Future intervention scenarios implemented from 2019 (MDA) or 2021 (vector control) are depicted by different colored lines as indicated in the legend

# Pre-intervention microfilarial prevalence of 80%

**Figure 6.1** Simulated dynamics between 2015 and 2035 in the microfilarial prevalence among individuals aged ≥ 5 years in settings with a 80% pre-control prevalence and no history of prior control. Future intervention scenarios implemented from 2019 (mass drug administration) or 2021 (vector control) are depicted by different colored lines as indicated in the legend.

**Figure 6.2** Simulated dynamics between 2015 and 2035 in the microfilarial prevalence among individuals aged ≥ 5 years in settings with a 80% pre-control prevalence and a 5-year history of annual mass drug administration (MDA). Future intervention scenarios implemented from 2019 (MDA) or 2021 (vector control) are depicted by different colored lines as indicated in the legend.

**Figure 6.3** Simulated dynamics between 2015 and 2035 in the microfilarial prevalence among individuals aged ≥ 5 years in settings with a 80% pre-control prevalence and a 10-year history of annual mass drug administration (MDA). Future intervention scenarios implemented from 2019 (MDA) or 2021 (vector control) are depicted by different colored lines as indicated in the legend.

**Figure 6.4** Simulated dynamics between 2015 and 2035 in the microfilarial prevalence among individuals aged ≥ 5 years in settings with a 80% pre-control prevalence and a 15-year history of annual mass drug administration (MDA). Future intervention scenarios implemented from 2019 (MDA) or 2021 (vector control) are depicted by different colored lines as indicated in the legend.

**Figure 6.5** Simulated dynamics between 2015 and 2035 in the microfilarial prevalence among individuals aged ≥ 5 years in settings with a 80% pre-control prevalence and a 20-year history of annual mass drug administration (MDA). Future intervention scenarios implemented from 2019 (MDA) or 2021 (vector control) are depicted by different colored lines as indicated in the legend.

**Figure 6.6** Simulated dynamics between 2015 and 2035 in the microfilarial prevalence among individuals aged ≥ 5 years in settings with a 80% pre-control prevalence and a 5-year history of biannual mass drug administration (MDA). Future intervention scenarios implemented from 2019 (MDA) or 2021 (vector control) are depicted by different colored lines as indicated in the legend.
